# Brain‐Targeted Cas12a Ribonucleoprotein Nanocapsules Enable Synergetic Gene Co‐Editing Leading to Potent Inhibition of Orthotopic Glioblastoma

**DOI:** 10.1002/advs.202402178

**Published:** 2024-06-28

**Authors:** Weimin Ruan, Sen Xu, Yang An, Yingxue Cui, Yang Liu, Yibin Wang, Muhammad Ismail, Yong Liu, Meng Zheng

**Affiliations:** ^1^ Henan‐Macquarie University Joint Centre for Biomedical Innovation Henan Key Laboratory of Brain Targeted Bio‐nanomedicine Henan International Joint Laboratory of Nanobiomedicine School of Life Sciences Henan University Kaifeng Henan 475004 China; ^2^ Henan Provincial Engineering Center for Tumor Molecular Medicine School of Basic Medical Science Henan University Kaifeng Henan 475004 China; ^3^ School of Ophthalmology and Optometry, School of Biomedical Engineering Wenzhou Medical University 270 Xuanyuanxi Road Wenzhou Zhejiang 325027 China

**Keywords:** Cas12a, CRISPR, gene‐editing, glioblastoma, nanocapsule

## Abstract

Gene‐editing technology shows great potential in glioblastoma (GBM) therapy. Due to the complexity of GBM pathogenesis, a single gene‐editing‐based therapy is unlikely to be successful; therefore, a multi‐gene knockout strategy is preferred for effective GBM inhibition. Here, a non‐invasive, biodegradable brain‐targeted CRISPR/Cas12a nanocapsule is used that simultaneously targeted dual oncogenes, *EGFR* and *PLK1*, for effective GBM therapy. This cargo nanoencapsulation technology enables the CRISPR/Cas12a system to achieve extended blood half‐life, efficient blood‐brain barrier (BBB) penetration, active tumor targeting, and selective release. In U87MG cells, the combinatorial gene editing system resulted in 61% and 33% knockout of *EGFR* and *PLK1*, respectively. Following systemic administration, the CRISPR/Cas12a system demonstrated promising brain tumor accumulation that led to extensive *EGFR* and *PLK1* gene editing in both U87MG and patient‐derived GSC xenograft mouse models with negligible off‐target gene editing detected through NGS. Additionally, CRISPR/Cas12a nanocapsules that concurrently targeted the *EGFR* and *PLK1* oncogenes showed superior tumor growth suppression and significantly improved the median survival time relative to nanocapsules containing single oncogene knockouts, signifying the potency of the multi‐oncogene targeting strategy. The findings indicate that utilization of the CRISPR/Cas12a combinatorial gene editing technique presents a practical option for gene therapy in GBM.

## Graphical abstract

A biodegradable brain‐targeted CRISPR/Cas12a nanocapsule (ANC@RNP) simultaneously targeted *EGFR* and *PLK1* for effective GBM therapy. ANC@RNP achieved extended blood half‐life, efficient blood‐brain barrier (BBB) penetration, active tumor targeting, and selective release. ANC@RNP lead to robust combinatorial gene editing and showed superior tumor growth suppression in U87MG and patient‐derived GSC orthotopic xenograft mouse models with negligible off‐target gene editing.

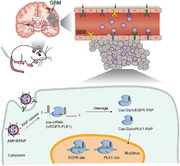

## 1 Introduction

Glioblastoma (GBM) is the most aggressive type of primary brain tumor with a poor prognosis.^[^ ^1^ ^]^ Multiple approaches have been applied for GBM treatment; however, due to complex pathogenesis, monotherapy has resulted in only marginal improvements in the median survival of GBM patients. Recently, combinatorial strategies such as multi‐chemotherapy,^[^ ^2^ ^]^ multiplexed RNA interfering (RNAi) therapy,^[^ ^3^ ^]^ and chemo‐RNAi therapy^[^ ^4^ ^]^ have shown great advancements in GBM treatment relative to the monotherapy approach, by targeting multiple genes, thereby inducing synergistic activity. However, chemo‐combinatorial therapeutic strategies usually lead to drug resistance and toxic side effects, while RNAi‐ combinatorial therapy induces only transient therapeutic effects.^[^ ^5^ ^]^ In this regard, a sustainable, safe, and drug‐resistance‐free therapeutic approach is highly desirable.

The clustered, regularly interspaced, short palindromic repeat (CRISPR)‐associated (Cas) system has emerged as a promising alternative treatment strategy in the last decade.^[^ ^6^ ^]^ CRISPR/Cas is a versatile RNA‐guided DNA editing tool that has been recently leveraged as an efficient tool for targeting genomic loci.^[^ ^7^ ^]^ The CRISPR‐based DNA editing technique could lead to indefinitely sustained oncogene knockout, successfully modulating the expression of functional proteins and outperforming other prevailing RNAi and conventional drugs that require continuous administration. As the most widely used CRISPR system, CRISPR/Cas9 has gained significant prominence, and several CRISPR/Cas9 systems have entered clinical trials.^[^ ^8^ ^]^ However, the single guide RNA (gRNA) for Cas9 provides the ability to edit individual genes, but multiplexed gene editing requires simultaneous or sequential delivery of multiple gRNAs, which greatly increases the risk of unwanted off‐target editing, leading to further genotoxicity concerns.

CRISPR/Cas12a (formerly Cpf1) is a generation of gene editing tool and is the second most widely used CRISPR system for gene knockout, which has found application primarily for therapeutic editing applications in vivo.^[^ ^9^ ^]^ Cas12a holds several advantages over Cas9 systems; it causes fewer off‐target cleavages and genotoxicity than Cas9 protein due to its lower potential for proto‐spacer mismatches.^[^ ^10^ ^]^ In addition, Cas12a only cuts target DNA via a single mature crRNA (42 nt), reducing the cost of chemical synthesis and in vitro transcription of crRNA.^[^ ^11^ ^]^ Moreover, Cas12a is capable of simultaneous editing at multiple genome loci with a single crRNA array, while exhibiting comparable efficiency to individual crRNA.^[^ ^12^ ^]^ These characteristics imply that Cas12a is a more powerful and safe gene editing tool relative to Cas9 in treating GBM via a combinatorial gene engineering strategy.

Despite the potential of CRISPR/Cas12a, its application has not yet been explored in GBM due to the sensitivity of CRISPR/Cas systems to blood enzymes, lack of blood‐brain barrier (BBB) permeability, and poor cellular uptake.^[^ ^13^ ^]^ Recently, both viral and non‐viral vectors have been explored to deliver CRISPR/Cas tools for in vitro and in vivo genome editing in therapeutic applications.^[^ ^14^ ^]^ Adeno‐associated viral (AAV) vectors are the most frequently used vector for in vivo gene editing research applications with CRISPR. However, long‐term expression of genome‐editing biomolecules with AAVs may expose patients to undesired off‐target editing or immune response.^[^ ^15^ ^]^ Thus, viral vectors pose safety concerns for therapeutic genome editing.^[^ ^14^ ^]^ Non‐viral vectors, such as lipids,^[^ ^16^ ^]^ polymers,^[^ ^17^ ^]^ DNA nanoclews,^[^ ^18^ ^]^ and biodegradable nanocapsules^[^ ^19^ ^]^ have been widely used to deliver CRISPR/Cas to achieve high editing efficiency and bio‐safety in vivo. Among them, biodegradable nanocapsules are highly promising because of their simple fabrication, high drug loading, biocompatibility, small particle size, and at‐site release ability.

**Figure 1:**
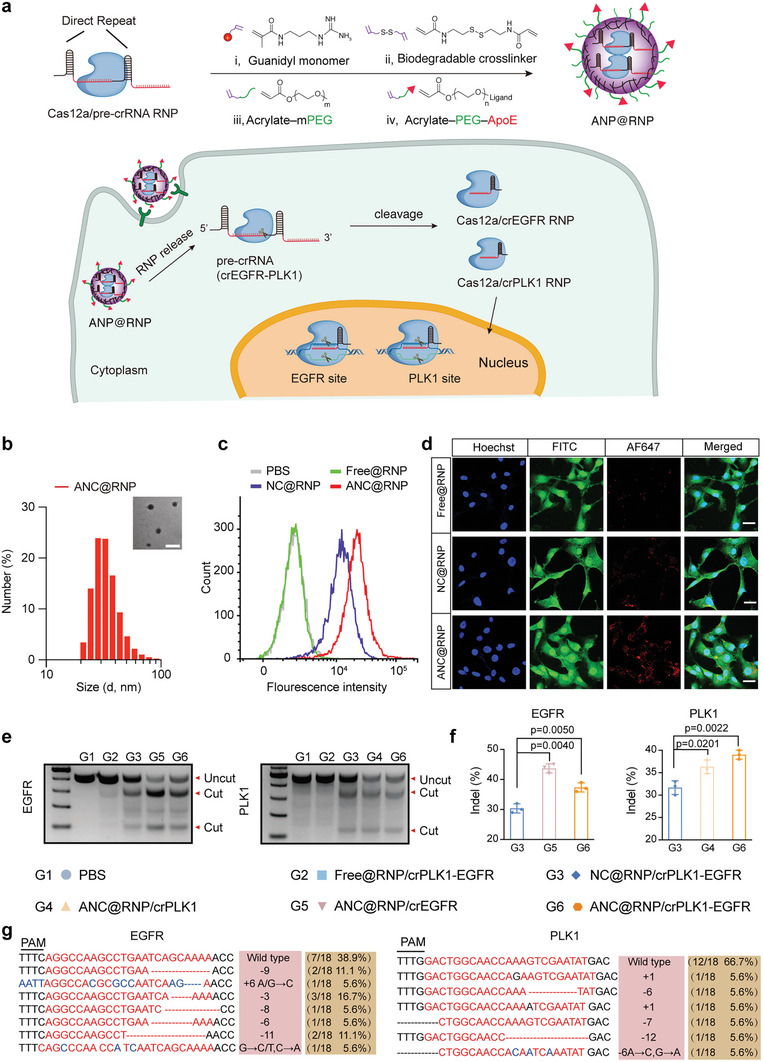
Preparation, in vitro delivery, and gene editing of ANC@RNP. a) Depiction of the self‐assembly process of the covalently crosslinked Cas12a nanocapsules (ANC@RNP) by in situ free‐radical polymerization and schematic illustrating multiplex gene editing with Cas12a and pre‐crRNA in cells. b) Size distribution and TEM image of ANC@RNP. c) Cell uptake of Alexa Fluor 647 (AF647)‐labeled ANC@RNP in glioblastoma U87MG cells by flow cytometry. d) CLSM imaging for AF647‐labeled ANC@RNP after 6 h of incubation with U87MG cells. In panel d, images from left to right are stained with Hoechst (blue), phalloidin‐FITC (green), AF647‐Cas12a (red), and an overlay of the corresponding images. Scale bar, 20 µm. e) Indels of *EGFR* and *PLK1* genes in U87MG cells transfected with ANC@RNP/crEGFR‐PLK1 or controls for 48 h and f) with quantification. g) Sanger sequencing results of PCR amplicons after EGFR and PLK1 gene editing in U87MG cells after treatment with ANC@RNP/crEGFR‐PLK1.

Here, we created an effective nanocapsule‐based CRISPR/Cas12a nanomedicine selectively targeting brain tumors for in vivo GBM therapy by utilizing Cas12a's multiplexed gene editing capabilities, which allow for combinatorial targeting via a single crRNA (84 nt). To our knowledge, this study marks the first‐time utilization of CRISPR/Cas12a gene editing system for the in vivo therapy of brain disease. Our Cas12a RNP gene‐editing nanosystem (abbreviated as ANC@RNP, **Figure** **1**a) achieved up to 30–60% double knock‐out of *EGFR* and *PLK1* oncogenes in glioma cell lines. Moreover, by targeting *EGFR* and *PLK1* oncogenes, the Cas12a RNP brain‐targeting nanocapsule system achieved higher anti‐tumor activity both in glioma cell lines in vitro and orthotopic xenografts models in vivo, exhibiting superior synergistic inhibition without inducing off‐target editing or systemic toxicity to normal tissues.

## 2 Results

### 2.1 Dual Gene Editing of Oncogenes by Cas12a/crRNA RNP

We first investigated whether CRISPR/Cas12a (*Acidaminococcus sp*. Cas12a, AsCas12a) could mediate multiple gene editing in the form of ribonucleoprotein with pre‐crRNA (RNP). We initially tested the performance of Cas12a RNP in the GBM cell line U87MG, selecting *EGFR* and *PLK1*, as suppressing these genes has been proposed as a potent synergic therapy. We analyzed the genomic sequence of *EGFR* (NM_0 05228) and *PLK1* (NM_0 05030) via the Crispor online database (http://crispor.tefor.net) and selected three high‐ranked crRNAs for each gene.

To identify crRNA candidates for further investigation, we employed the lipoCRISPRmax for RNP transfection. The Cas12a/crRNA RNP editing efficiency was measured 48 h post lipoCRISPRmax transfection for each crRNA (30 nm). T7E1 digestion assays showed the indel efficiency of each gene was comparable to Cas9 RNP editing at the same concentration of RNP (Figure S1, Supporting Information). The crRNAs with the highest efficiency were then chosen for the subsequent pre‐crRNA preparation designed by protospacers linked with 20 nt direct sequence (DR). Next, we performed an in vitro cleavage assay to examine the activity of Cas12a‐RNP complexes. Cas12a nuclease possesses inherent RNase activity and can generate multiple guide crRNAs from a single pre‐crRNA transcript (Figure S2, Supporting Information).^[^ ^20^ ^]^ We found Cas12a‐mediated pre‐crRNA processing in vitro was incubation‐time dependent, which is consistent with a previous report.^[^ ^11^ ^]^ In the cleavage assay, 7.5 nm pre‐crRNA was cleaved with an equal amount of Cas12a protein. Data suggested that the Cas12a‐forming‐RNP began processing pre‐crRNA (4 crRNA linked, 164 nt) into single crRNA (42 nt) in 10 min and completely converted pre‐crRNA to mature crRNA in 20 min (Figure S3, Supporting Information). These data may reflect the sequential molecular events after transfection, that delivered cytoplasmic Cas12a RNase first cut pre‐crRNAs, releasing individual single crRNAs which then enter the nucleus driven by the nuclear localization signal (NLS), mediating editing of each gene site (Figure 1a). We then assessed the indel rates of pre‐crRNA (crEGFR‐PLK1) in U87MG cells compared to individual crRNA. T7E1 assay showed that pre‐crRNA induced comparable indel levels (38% and 34%) to that mediated by individual crRNAs (34% and 32%) at the same dose of Cas12a and crRNA (30 nm) (Figure S3, Supporting Information). Collectively, these data demonstrate that Cas12a/pre‐crRNA RNP represents an effective system for manipulating synergic anti‐tumor genes.

### 2.2 Preparation of ANC@RNP and Evaluation of In Vitro Delivery

Efficient delivery systems are crucial for effective gene editing due to the intricate nature, sensitivity, and limited cell uptake or targeting ability of the CRISPR/Cas12a system. Recently published work indicated that small nanocapsules efficiently transported Cas9/gRNA in vitro and in vivo.^[^ ^21^ ^]^ Given that Cas9/gRNA and Cas12a/crRNA RNP share similar characteristics, we expanded the nanocapsules delivery strategy for Cas12a/crEGFR‐PLK1 RNP system and evaluated its potential in GBM suppression. To facilitate BBB penetration, we grafted Apolipoprotein E (ApoE) peptide onto the surface of our nanocapsules, which further expedites BBB transcytosis and tumor targeting of Cas12a RNP.^[^ ^22^ ^]^ The ApoE‐decorated nanocapsules were fabricated via in situ polymerization, with encapsulation of Cas12a/crRNA RNP yielding the final nanosystem ANC@RNP.^[^ ^21^ ^]^ The physicochemical properties of ANC@RNP, such as complexation ability, particle size, and zeta potential, were assessed by dynamic light scattering (DLS) and transmission electronic microscopy (TEM). DLS results revealed that the average hydrodynamic diameter of ANC@RNP was ≈30 nm, exhibiting uniform distribution comparable to that of non‐targeted NC@RNP. In comparison, the size of bare Cas12a/crRNA was 11 nm. The zeta potential of ANC@RNP showed a slight positive, +7–9 mV, in comparison naked Cas12a/crRNA showed a negative zeta potential at ‐22 mV (Figure 1b; Table S1, Supporting Information). The nanostructure of ANC showed spherical morphology as determined by TEM images (Figure 1b). Moreover, the ANC@RNP nanocapsules were stable under the physiological (37 °C) and storage (4 °C) conditions over 1 week and 1 month, respectively (Figure S4, Supporting Information). These results demonstrated that ANCs could accommodate charge heterogeneity of Cas12a/crRNA (84 nt) RNP to form a covalently stabilized RNP nanomedicine.

To determine whether Cas12a/crRNA RNP could be effectively delivered into U87MG cells, we used flow cytometry and confocal laser scanning microscopy (CLSM) to track the uptake efficiency and intracellular distribution of Cas12a protein that had been labeled with the Alexa Fluor 647 (AF647). Nanocapsules with ApoE‐decoration, ANC@RNP, displayed 2.3‐fold higher cellular uptake compared to non‐ApoE functionalized NC@RNP nanocapsules as quantitatively measured by flow cytometry in U87MG cells (Figure 1c). CLSM observation further validated that ANC@RNP showed greater endocytosis into U87MG cells than NC@RNP nanocapsules after a 6 h incubation (Figure 1d). In contrast, free Cas12a RNP showed negligible entry into U87MG cells. Importantly, even at high concentrations (200 nm), ANC@RNP with unspecific crRNA did not induce cytotoxicity in U87MG cells (2% cell death) as determined by CCK8 assays (Figure S5, Supporting Information). These findings show that ANC nanocapsules can efficiently deliver Cas12a RNP into glioblastoma cells while having minimal effects on cellular proliferation.

### 2.3 Synergistic In Vitro Gene Editing by ANC@RNP

Next, the gene editing potency of ANC@RNP to target *EGFR* and *PLK1* was assessed through dual insertion/deletion (indel) in U87MG cells at the equivalent RNP doses. T7E1 digestion assay results demonstrated that ANC@RNP achieved more potent activity at *EGFR* and *PLK1* gene sites versus non‐targeted NC@RNP after 48 h incubation in U87MG cells (Figure 1e,f), signifying the importance of ApoE‐ligand in tumor targeting. Dual‐crRNA pairs targeting *EGFR* or *PLK1* had similar gene editing efficiency compared to individual *EGFR* or *PLK1* controls (Figure 1e,f). Unsurprisingly, the non‐encapsulated free Cas12a RNP treatment control group exhibited minimal indel in the T7E1 assay, indicating the efficiency of nanocapsule delivery. Consistent with the T7E1 assay, Sanger sequencing results after subcloning of these PCR amplicons also confirmed the gene editing ability of ANC@RNP/crEGFR‐PLK1, which achieved 61% and 33% gene knock‐out of *EGFR* and *PLK1* target genes, respectively (Figure 1g).

**Figure 2:**
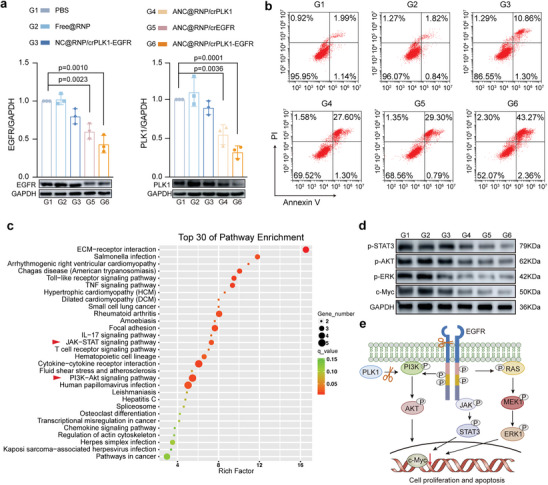
Synergistic effect of ANC@RNP/crEGFR‐PLK1. a) Quantitation of Western Blot of EGFR and PLK1 expression relative to GAPDH after transfection with ANC@RNP/crEGFR‐PLK1 in U87MG cells. b) Apoptosis induction in U87MG cells after 72 h incubation with ANC@RNP/crEGFR‐PLK1 or controls at equivalent doses of Cas12a. c) Pathway enrichment of differentially expressed genes in U87MG cells after ANC@RNP/crEGFR‐PLK1 treatment. The red arrow highlights enriched pathways. d) Western Blot of EGFR and PLK1 down‐stream signal expression relative to GAPDH. Quantitation analysis is presented in Figure S8 (Supporting Information). e) Depiction shows the synergistic effect of EGFR and PLK1 on downstream signals.

The anti‐tumor properties of combinatorial ANC@RNP/crEGFR‐PLK1 were then tested in vitro. First, we conducted Western Blot to assess the protein level of EGFR and PLK1 in U87MG cells. ANC@RNP/crEGFR‐PLK1 treatment reduced EGFR and PLK1 protein expression in U87MG cells by 67% and 56%, respectively (**Figure** **2**a). These results demonstrated that the ANC@RNP system could efficiently mediate dual gene targeting and editing at the protein level, validating the success of our multi‐knockout gene strategy. Next, the induction of cellular apoptosis was investigated by flow cytometry using the Annexin‐V and PI staining methods. These results revealed that combined targeting of EGFR and PLK1 synergistically promoted late and early apoptosis (45.6%) in U87MG cells, compared to either *EGFR* (31.1%) or *PLK1* (28.9%) targeting alone (Figure 2b). It is worth noting that ANC@Cas12a/crEGFR‐PLK1 treatment also reduced colony formation number, which was considerably lower than that shown by NC@Cas12a/crEGFR‐PLK1 or other treatment controls (Figure S6, Supporting Information), which aligned with effects on cell proliferation and apoptosis induction.

To further identify the potential molecular mechanism for the synergistic effect of ANC@Cas12a/crEGFR‐PLK1, we performed transcriptome analysis by bulk RNA sequencing in U87MG cells. Heat map analysis showed that the ANC@Cas12a/crEGFR‐PLK1 treatment had a greater impact on gene expression than the individual crRNA controls (Figure S7a–c, Supporting Information). Differentially expressed genes (DEGs) were identified based on a false discovery rate (FDR) threshold of 0.05. GO (Gene Ontology) functional annotation of differential gene enrichment revealed that ANC@Cas12a/crEGFR‐PLK1 treatment up‐regulated genes associated with programmed cell death and autophagy while down‐regulating genes involved in cell proliferation and cell division. The largest number of substantially up‐regulated genes was found in the PI3K‐Art and JAK‐STAT signaling pathway, as determined by KEGG pathway mapping (Figure 2c), which has been reported as a hallmark of many cancers as it orchestrates the tumor microenvironment, cell survival, metastasis, and metabolism.^[^ ^23^ ^]^ It has also been suggested that the combined inhibition of phosphorylation‐MEK and PI3K‐Akt signaling might enhance the inhibition of tumor cell growth.^[^ ^24^ ^]^ To verify the RNA sequencing analysis results, we further evaluated the mRNA level of the PI3K‐Art signal‐related genes, LAMA 5 and TNXB, and the JAK‐STAT signal‐related genes, IL7R and STAT3. Quantitative PCR analysis results confirmed the down‐regulation of these pathways (Figure S8, Supporting Information). Next, we tested whether the core downstream genes of PI3K‐Akt and JAK‐STAT axis were impacted, which might further elaborate the mechanism of the synergistic anti‐tumor activity mediated by ANC@RNP/crEGFR‐PLK1. We found that expression of the down‐stream PI3K‐Akt and JAK‐STAT proteins, including phosphorylated STAT3, AKT, and ERK were potently decreased in U87MG cells after ANC@RNP/crEGFR‐PLK1 treatment (Figure 2d; Figure S7, Supporting Information). In addition, oncogene c‐Myc was synergistically reduced by EGFR and PLK1 deletion (Figure 2d), similar to the effects of previously described pharmacological inhibitors.^[^ ^25^ ^]^ In light of these findings, we propose a model to better explain the synergistic effect of *EGFR* and *PLK1* deletion in this study (Figure 2e). These findings suggested that ANC@RNP could be used as a versatile multiple‐gene knock‐out tool to promote synergistic in vivo therapy of glioblastoma by simultaneous targeting of *EGFR* and *PLK1*.

### 2.4 In Vivo Pharmacokinetics, Biodistribution, and Glioma Targeting

**Figure 3:**
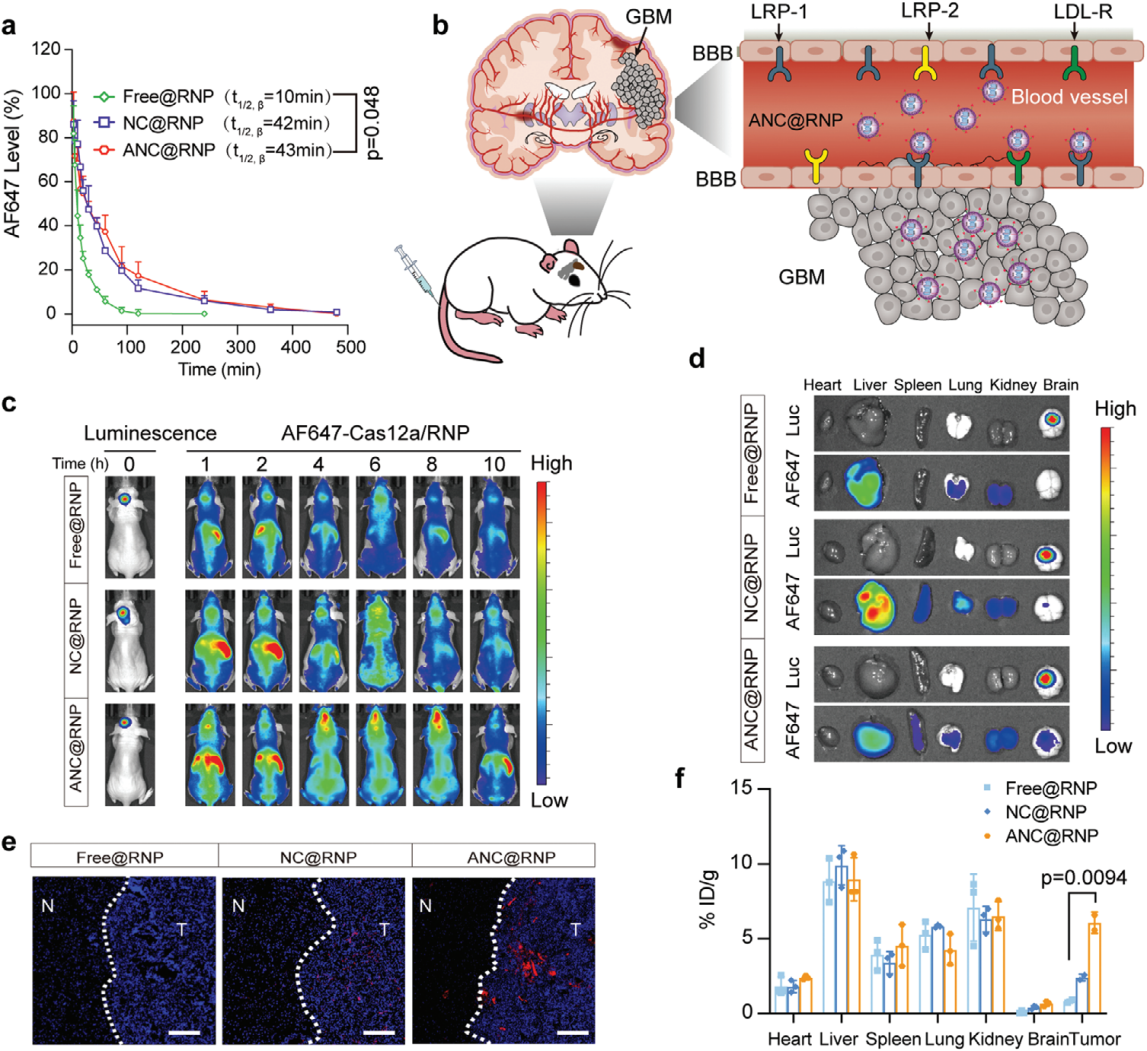
In vivo pharmacokinetics, tumor targeting and distribution. a) Pharmacokinetics of ANC@RNP and controls in tumor‐free mice (1.5 mg Cas12a equiv./kg). Cas12a was labeled with AF647. Data are mean ± SD (*n* = 3). b) Schematic illustration of ANC@RNP of the brain tumor targeting mechanism. c) Whole body fluorescence images of orthotopic U87MG‐Luc human glioblastoma tumor‐bearing nude mice at different time points following injection of ANC@RNP or controls (1.5 mg Cas12a equiv./kg). d) Ex vivo fluorescence images of major organs taken from nude mice bearing orthotopic U87MG‐Luc human glioblastoma tumor after 8 h *i.v*. injection of ANC@RNP or controls (1.5 mg Cas12a equiv./kg). e) Confocal imaging of the localization of ANC@RNP and controls in excised brain tissues. Blue, Hoechst; Red, AF647‐Cas12a. N: normal brain tissue, T: tumor. Scale bars: 50 µm. f) Quantitation of AF647‐Cas12a accumulation in different organs. AF647‐Cas12a levels were determined by fluorescence spectroscopy and are expressed as % ID/g. Data are presented as mean ± SD (*n* = 3).

Next, we assessed the in vivo pharmacokinetics, biodistribution, and glioma targeting to predict the in vivo performance of ANC@RNP. For pharmacokinetics, AF647‐Cas12a‐loaded ANC@RNP was *i.v*. injected into healthy Balb/c mice (without tumor), followed by blood collection to quantify fluorescence. ANC@RNP and NC@RNP demonstrated similarly prolonged elimination half‐lives (t_1/2, β_) of 43.0 and 42.0 min, respectively, which were significantly longer than that of free RNP (t_1/2, β_ = 10 min; **Figure** **3**a). These findings indicated that nanocapsules protected Cas12a RNP from degradation, thereby sustaining their circulation in the bloodstream. We next assessed the BBB penetration ability of the ApoE‐modified nanocapsules in an in vitro BBB transwell model as reported previously.^[^ ^26^ ^]^ The transwell system was developed using endothelial cells (mouse bEnd.3 cells) cultured in the upper insert (Figure S9, Supporting Information). The transport ratio was calculated as a function of fluorescence intensity measured at pre‐determined time points (1, 2, 6, 24, and 48 h). Relative to non‐ApoE decorated NC@RNP nanocapsules, ApoE decorated ANC@RNP resulted in much higher AF647‐Cas12a signal intensity indicating significantly higher traversal across the BBB‐barrier (Figure S9b, Supporting Information), emphasizing the importance of ApoE shuttle ligand in receptor (LRP1, LRP2, LDLR)‐mediated transcytosis of BBB cells (Figure 3b).^[^ ^22^ ^]^

The in vivo BBB permeability and intracranial glioma targeting ability of ANC@RNP was then assessed by *i.v*. injection of 1.5 mg kg^−1^ equivalent AF647‐labeled RNP in orthotopic U87MG‐Luc‐bearing nude mice (Figure 3b). Using a fluorescence imaging system (IVIS, PerkinElmer), the biodistribution of nanocapsules at different time points after injection was tracked. At 8 h after administration, ANC@RNP treated mice had the strongest AF647 fluorescence intensity in the brain, outperforming the non‐ApoE modified NC@RNP and free RNP controls (Figure 3c). To further assess the biodistribution of the nanocapsules, U87MG‐Luc glioma‐bearing mice were sacrificed 8 h after *i.v*. injection, and the brains and major organs were excised and imaged. Unsurprisingly, brains from the ANC@RNP‐treated mice showed higher RNP accumulation (Figure 3d). Importantly, IVIS *ex vivo* imaging revealed that the fluorescence of ANC@RNP in the brain was significantly co‐localized with the luminescence of orthotopic tumor (Figure 3d), which could suggest the targeting ability of the modified ApoE ligand. The ability of ANC@RNP to traverse BBB and target gliomas in vivo was then studied in brain slices using confocal imaging (Figure 3e). After 8 h, there was a weak accumulation of free RNP in the glioma tissue of mice, while mice receiving non‐ApoE functionalized NC@RNP showed mild fluorescence. However, a bright red fluorescence was seen in the tumor tissues of mice treated with ANC@RNP, again demonstrating the significance of ApoE‐functionalization in mediating BBB permeability and GBM targeting. Quantification on the distribution of ANC@RNP in various organs showed that brain tumor accumulation of ANC@RNP increased to 6.1% of injected dose per gram of tissue (% ID/g), which was 2.7‐ and 7.3‐fold higher than treatment with NC@RNP or free RNP control, respectively (Figure 3f). We also evaluated glioma tumor tissue penetration using a 3D U87MG tumor spheroid model. As expected, ANC@RNP showed the deepest tumor spheroid penetration capability (Figure S10, Supporting Information), providing a solid foundation for treating intracranial GBM in vivo. Collectively, these results demonstrate that Cas12a RNP can be administered into glioma tissue cells using nanocapsules, leading to effective glioma penetration and accumulation.

### 2.5 Anti‐Tumor Potential of ANC@RNP in Orthotopic Glioblastoma Xenograft Model

**Figure 4:**
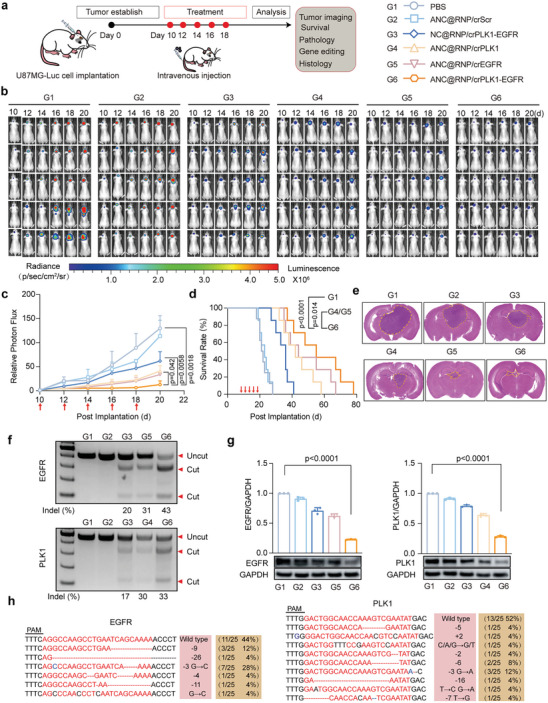
ANC@RNP mediated dual gene editing and anti‐tumor activity in orthotopic GBM xenografts. a) Schematic showing the timeline of the orthotopic tumor treatment. b) Luminescence images of orthotopic U87MG‐Luc human glioblastoma tumor‐bearing nude mice following treatment with ANC@RNP/crEGFR‐PLK1, ANC@RNP/crEGFR, ANC@RNP/crPLK1, NC@RNP/crEGFR‐PLK1, ANC@RNP/crScr, or PBS. Mice were *i.v*. injected at a dose of 1.5 mg Cas12a equiv./kg on days 10, 12, 14, 16, and 18 after tumor implantation. c) Quantified luminescence levels of mice using the Lumina IVIS III system following the indicated treatments (*n* = 5). d) Mean mouse survival was monitored and analyzed by Kaplan‐Meier plots. e) H&E staining of whole brain tissue excised on day 20 from euthanized U87MG‐Luc tumor‐bearing mice treated with different nanocapsule formulations as described above. The yellow dotted line represents the boundary between the tumor and normal brain tissue. f) Indel frequency of *EGFR* and *PLK1* genes in tumor tissues excised from mice on day 20. g) Western Blot and quantitation of EGFR and PLK1 protein expression in tumor tissues excised on day 20; GAPDH was used as a reference. Data are presented as mean ± SD (*n* = 3). h) Sanger sequencing results of PCR amplicon clone of EGFR and PLK1.

The next step was to determine whether the high ANC@RNP/crEGFR‐PLK1 genome editing rates seen in vitro would translate to in vivo therapeutic efficacy. Using the U87MG‐Luc orthotopic glioblastoma xenograft model, mice were divided randomly into six groups. After 10 days post‐implantation of U87MG‐Luc cells, mice were systemically administered with ANC@RNP/crEGFR‐PLK1, ANC@RNP/crEGFR, ANC@RNP/crPLK1, NC@RNP/crEGFR‐PLK1, ANC@RNP/crScr, or PBS via the tail‐vein every 2 days for a total of 5 cycles (**Figure** **4**a). Bioluminescence intensity imaging was used to compare the tumor progression of mice receiving the various treatments. Mice treated with targeted ANC@RNP/crEGFR‐PLK1 nanocapsules exhibited potent inhibition of tumor growth after five RNP doses, as evidenced by showing the weakest bioluminescence intensity (Figure 4b,c). In contrast, aggressive tumor growth was seen in the mice receiving ANC@RNP/crScr or PBS, which showed no tumor inhibition. Interestingly, targeted ANC@RNP/crEGFR‐PLK1 therapy resulted in significant tumor growth reduction when compared to single gene editing counterparts (ANC@RNP/crEGFR or ANC@RNP/crPLK1; Figure 4b,c), indicating the synergistic role of dual EGFR and PLK1 inhibition in tumor progression. Unsurprisingly, the lifespan of mice treated with combinational ANC@RNP/crEGFR‐PLK1 showed the highest increase in median survival (55 days) compared to mice treated with the single gene editing therapies ANC@RNP/crEGFR (44 days) or ANC@RNP/crPLK1 (both 42 days) or non‐targeted NC@RNP/crEGFR‐PLK1 treatment (36 days; Figure 4d). It is important to mention that our innovative dual‐gene deletion technology (ANC@RNP/crEGFR‐PLK1) achieved a median survival period of 55 days, which is significantly higher than the 32 days reported with the typical clinical standard temozolomide (TMZ) in our prior examination on GBM treatment.^[^ ^2a^ ^]^ The size of excised glioma tissue taken from mice treated with ANC@RNP/crEGFR‐PLK1 was consistently smaller than tumor tissue taken from controls (Figure 4e).

T7E1 digestion assay and Sanger sequencing test were then used to determine indel frequency and validate in vivo gene editing following ANC@RNP/crEGFR‐PLK1 therapy. Mice receiving the combinational ANC@RNP/crEGFR‐PLK1 showed high gene editing levels of EGFR (43%) and PLK1 (33%) (Figure 4f), which was similar or higher to single crRNA treatment, but much higher than non‐targeted NC@RNP/crEGFR‐PLK1 treatment. We also quantified the protein expression levels of EGFR and PLK1 in the dissected glioma tissues by Western Blot. These results demonstrated that EGFR and PLK1 expression decreased by ≈78% and ≈76%, respectively, after treatment with ANC@RNP/crEGFR‐PLK1 (Figure 4g). Sanger sequencing after subcloning of PCR amplicons further confirmed the indel of both target genes (Figure 4h). Immunohistochemical (IHC) staining was then used to assess the induction of apoptosis in tumor tissue. In comparison to control groups, IHC staining indicated that ANC@RNP/crEGFR‐PLK1 treatment dramatically reduced the proliferation marker Ki67 but greatly increased the expression of the tumor apoptosis markers Caspase‐3 and TUNNEL (Figure S11, Supporting Information). These results showed that ANC@RNP/crEGFR‐PLK1 treatment can efficiently mediate multiplexed gene editing to decrease glioma growth in vivo, indicating a promising potential for glioma treatment.

### 2.6 In Vivo Therapeutic Efficacy in Mice Xenografts of Patient‐Derived Glioma Stem Cells

**Figure 5:**
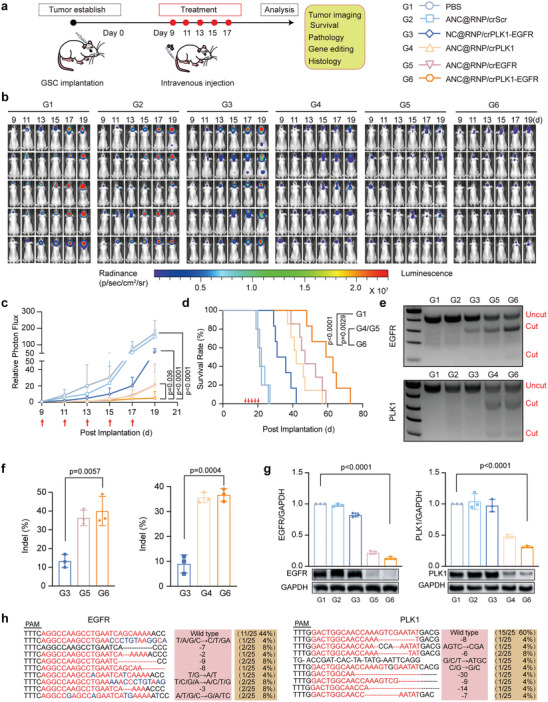
ANC@RNP mediated dual gene editing and anti‐tumor activity in orthotopic GSC xenografts. a) Schematic diagram shows the establishment of the patient‐derived GSC xenograft model and treatment schedule. b) Luminescence images of orthotopic GSC xenograft glioblastoma tumor‐bearing nude mice following treatment with ANC@RNP/crEGFR‐PLK1 and controls. Mice were *i.v*. injected at a dose of 1.5 mg Cas12a equiv./kg on day 9, 11, 13, 15, and 17 after tumor implantation. c) Quantitative analysis of bioluminescence images after treatment as depicted in (b). Data are shown as the mean ± SD, *n* = 5. d) The mean survival of mice was monitored and analyzed by Kaplan‐Meier plots. e) Indel frequency of EGFR and PLK1 gene in tumor tissues excised from mice on day 19 after tumor implantation. f) Quantitative analysis of gene indels of *EGFR* and *PLK1* from three replicates. g) Western Blot and quantitation of EGFR and PLK1 protein expression in tumor tissues excised on day 19 after tumor implantation. GAPDH was used as a reference. Data are mean ± SD (*n* = 3). h) Sanger sequencing results of PCR amplicons’ clone of *EGFR* and *PLK1*.

We further explored the therapeutic potential of ANC@RNP/crEGFR‐PLK1 in an orthotopic patient‐derived xenograft (PDX) model constructed from patient‐derived glioma stem cells (GSCs).^[^ ^21^ ^]^ The use of GSCs‐based models better recapitulates the natural process of carcinogenesis, tumor heterogeneity, accelerated tumor growth, and the development of resistance to therapy.^[^ ^27^ ^]^ As a result, GSCs have emerged as a new therapeutic target in patients with glioblastoma. Here, we evaluated the therapeutic efficacy of ANC@RNP/crEGFR‐PLK1 treatment in a PDX mouse model. Nanocapsules were administered every 2 days, 9 days after GSCs implantation for 5‐cycles (**Figure** **5**a). Tumor progression was tracked by bioluminescence imaging as described above for the U87MG xenograft mouse model. Similarly to the tumor growth inhibition observed in the U87MG‐Luc model, treatment with ANC@RNP/crEGFR‐PLK1 potently inhibited PDX tumor growth compared to the single gene editing treatments ANC@RNP/crEGFR and ANC@RNP/crPLK1, the non‐targeting group NC@RNP/crEGFR‐PLK1 or PBS controls (Figure 5b–d). As a result, the median survival time of mice receiving ANC@RNP/crEGFR‐PLK1 was 61 days, which was significantly longer than that of individual crRNAs (47 days for crEGFR, 42 days for crPLK1), non‐targeted NC@RNP/crEGFR‐PLK1 (34 days) or PBS controls (21 days; Figure 5d).

To investigate gene editing in GSC cells, we next excised brain tumor tissue at the end of treatment and pooled them together for subsequent T7E1 detection assay and Sanger‐sequencing analysis. Accordingly, we detected ≈41% and ≈38% indel formation of EGFR and PLK1 after treatment with ANC@RNP/crEGFR‐PLK1 (Figure 5e,f). Moreover, compared to PBS controls, the protein levels of EGFR and PLK1 were lowered by 88% and 70%, respectively, showing the potent in vivo gene editing ability of ANC@RNP/crEGFR‐PLK1 treatment (Figure 5g). Next, indel results were validated by Sanger sequencing of brain tissue after ANC@RNP/crEGFR‐PLK1 treatment (Figure 5h). These results were consistent with the T7E1 assays. In summary, ANC@RNP generated specific synergistic dual gene editing in vivo in the PDX pre‐clinical mouse model forming the basis for potent inhibition of GBM progression.

### 2.7 In Vivo Biocompatibility and Off‐Target Analysis

**Figure 6:**
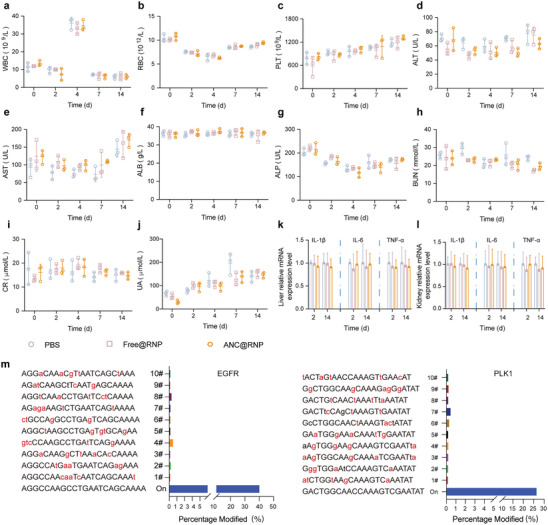
Safety evaluation of ANC@RNP. a–c) Analysis of routine blood parameters and d–j) blood biochemistry of healthy BALB/c mice treated with ANC@RNP, free RNP or PBS on day 2 or 14 after nanocapsule injection. Data are presented as mean ± SD (*n* = 3). k,l) mRNA expression of the pro‐inflammatory cytokines IL‐1β, IL‐6, and TNF‐α in the liver and kidney were assessed on days 2 and 14 after a single dose administration of ANC@RNP, free RNP, and PBS. Data are presented as mean ± SD (*n* = 3). m) Next‐generation sequencing analysis demonstrates off‐target indels in tumor cells dissected from U87MG‐bearing mice. Lowercase letters are mismatched nucleotides. Representative deep sequencing analysis demonstrating various insertion and deletion mutations are presented in Figure S14 (Supporting Information).

Biosafety of nanomedicines and the off‐target effects of Cas12a are major concerns for clinical translation. Thus, we first evaluated blood biochemistry parameters to ensure ANC@RNP was safe to administer. After a single injection (*i.v*.) of the ANC@RNP/crScr, free RNP or PBS into healthy Balb/c mice (*n* = 3), blood serum was obtained at various time points (at day 0, 2, 4, 7, and 14) for analysis. For hematological factors, we not only evaluated white blood cells (WBC), red blood cells (RBC) and platelet (PLT), but we also tested the alanine aminotransferase (ALT), aspartate aminotransferase (AST) and serum Albumin (ALB) to assess liver function with blood urea nitrogen (BUN), creatinine (CR) and uric acid (UA) to assess kidney function. No obvious changes in these parameters were seen after ANC@RNP treatment compared with PBS, indicating negligible side effects on the main organs of metabolism (**Figure** **6**a–j). Further, we used a quantitative RT‐PCR assay to see if ANC@RNP induced any immunological response in healthy Balb/c mice in vivo. On days 2 and 14 following treatment, ANC@RNP induced modest immunogenicity relative to PBS control groups, further confirming biosafety (Figure 6k,l). Moreover, the IgG assay and the corresponding hematoxylin‐eosin (H&E) histopathological examinations of critical organs after 2 weeks of ANC@RNP nanocapsules after single administration into healthy BALB/c mice further confirmed the safety of ANC@RNP, that showed negligible long‐term immunogenicity and no damage to critical organs (Figure S12, Supporting Information). In addition, major organs from the treated mice used in the U87MG model were sectioned and stained by H&E staining for histopathological analysis. These results showed no signs of damage to the lungs, heart, liver, spleen, or kidney resulting from ANC@RNP or PBS treatment (Figure S13, Supporting Information).

To find putative off‐target sites in human genomes, we used Cas‐OFFinder to identify top 10 off‐targets or mismatched sites in the human genome, which have 1–5 mismatched nucleotides of crEGFR and crPLK1 on‐sites (Table S7, Supporting Information).^[^ ^28^ ^]^ We applied targeted deep sequencing to see if these sites were cleaved in tumor tissues excised from the U87MG xenograft model. Next‐generation sequencing (NGS) results showed that ANCs@RNP/crEGFR‐PLK1 treatment induced indels at the two on‐target sites with a frequency of 40.1% and 26.3%, respectively (Figure 6m), which was consistent with T7E1 assay and Sanger sequencing (Figure 4e; Figure S14, Supporting Information). However, indel rates at the 1–5 base mismatched sites were less than 0.6%, which was below the detection limit (Figure 6m). It means negligible off‐target effect was found by NGS and Cas‐OFFinder predicted site validation. Next, off‐target efficiency was evaluated in the GSC xenograft model where no indels were found in tumor tissues excised from ANC@RNP/crEGFR‐PLK1 treated PDX mice (Figure S15, Supporting Information). Therefore, the delivery of Cas12a RNP via ANC nanocapsules is extremely selective while producing negligible off‐target effects in glioma cells.

## 3 Discussion

Glioblastoma is the deadliest malignant primary brain tumor that is highly resistant to current treatment modalities^[^ ^29^ ^]^ due to its highly diffuse infiltration and multiplex genetic alterations. *EGFR* and *PLK1* are the two most commonly overexpressed genes participating in a positive regulatory loop that contributes to GBM development.^[^ ^30^ ^]^ Several drug candidates have been developed to target these oncogenes; BI2536 and static are a combination of EGFR/PLK1 inhibitors that were demonstrated to induce better anti‐cancer effect relative to treatments that target either PLK1 or EGFR alone.^[^ ^25^ ^]^ Despite their significant promise, chemical inhibitors usually induce higher levels of toxic side effects and are subject to the development of multi‐drug resistance when applied as combination treatments.^[^ ^5^ ^]^

As an alternative to chemical inhibitors, CRISPR‐Cas systems are promising in cancer therapy. CRISPR‐Cas systems can be classified into Class 1 and Class 2 based on whether a multi‐ or single‐subunit Cas protein effector complex, respectively.^[^ ^31^ ^]^ Although class 2 systems such as Cas9/Cas12a have disadvantages in long‐range genome manipulations and deletion in eukaryotes, they showed superior editing efficiency and labor‐saving purification process than Class 1 systems.^[^ ^32^ ^]^ So, they have been widely utilized for human cell genome editing. The significance of CRISPR therapeutic genome editing technology can be judged from its application in clinical trials for sickle cell disease (ClinicalTrials.gov: NCT03745287), CAR‐T cell therapy (ClinicalTrials.gov: NCT03399448) and β‐thalassemia (ClinicalTrials.gov: NCT03655678). Although CRISPR therapeutic genome editing technology might allow good anti‐tumor effects without toxicity, its application to most types of solid tumors remains a great challenge, particularly for brain tumors, because of the low targeting specificity and potential for off‐target effects that might cause harm to healthy brain cells.^[^ ^33^ ^]^ CRISPR delivered using a technology designed specifically to target brain tumors, may help overcome these problems. We and others have used nanoparticles with excellent safety and high selectivity engineered to cross the blood‐brain barrier (BBB) for brain tumor targeting, boosting their effectiveness against GBM tumors.^[^ ^21^, ^34^ ^]^ For example, we have developed a nonviral CRISPR/Cas9 delivery system that uses peptide‐functionalized biodegradable nanocapsules to encapsulate and protect Cas9 protein and sgRNA for noninvasive targeted gene knockdown.^[^ ^21^ ^]^ However, due to the complexity of GBM pathogenesis, a single gene editing nanosystem mediated by CRISPR/Cas9 is insufficient to yield superior anti‐GBM outcomes.

To overcome this limitation, here we developed a dual gene editing nanosystem for treating GBM using effective delivery of CRISPR/Cas12a rather than CRISPR/Cas9, despite the fact that the CRISPR/Cas12a gene‐editing system has not yet been reported in vivo brain disease therapy. By design, our Cas12a/crRNA RNP nanomedicine features numerous distinctive elements: 1) it contains crEGFR and crPLK1 linked with the direct sequence for dual gene knockout; 2) higher Cas12a/pre‐crRNA RNP loading efficiency of nearly 100% and on‐demand release in tumor cells; 3) decoration with the BBB shuttle ligand ApoE‐peptide effectively guides the nanosystem toward precise brain tumor targeting to decrease the chance off‐target gene editing; 4) combinatory EGFR and PLK1 gene editing ability synergistically enhances potent in vivo anti‐tumor effects not before observed in CRISPR‐based gene therapy. We show that ANC@RNP/crEGFR‐PLK1 nanomedicine mediated efficient dual gene targeting of *EGFR* and *PLK1* oncogenes in glioma cells with enhanced anti‐tumor effect versus single crRNA targeting of oncogenes. Moreover, the nanosystem significantly improved the pharmacokinetics, BBB transcytosis, and brain tumor targeting of Cas12a/crRNA RNP, which led to the substantial inhibition of GBM progression resulting from the crEGFR and crPLK1 synergism. Given that CRISPR off‐target gene editing and other biosafety concerns are the major bottleneck limitations that seriously impede their clinical application, we critically evaluated the potential biosafety and off‐target effects of ANC@RNP. Biosafety evaluations showed that ANC@RNP exhibited minimal cytotoxicity in vitro and in vivo, with no indication of damage to major organs. Most importantly, the non‐invasive administration of ANC@Cas12a RNP caused negligible off‐target side effects (<0.5%) in both U87MG and PDX stem cell GBM xenograft models, which can be mainly ascribed to their stable encapsulation, precise targeting, and on‐demand release of Cas12a/crRNA RNP at the tumor site. Noted that, although the non‐viral nanocapsules avoid the immunological risks of viral vectors, the preexisting adaptive immunity of CRISPR‐Cas system still needs attention in future studies.^[^ ^15^, ^35^ ^]^

In summary, we reported a non‐invasive biodegradable brain‐targeted CRISPR/Cas12a nanocapsule to simultaneously target multiple oncogenes (EGFR and PLK1) for effective GBM treatment. This robust synergistic gene‐editing therapy strategy overcomes the therapeutic ineffectiveness of monotherapy, and avoids the drug resistance or transient effects mediated by chemotherapy and RNAi therapy, respectively. To the best of our knowledge, this is the first report leveraging CRISPR gene editing system for dual gene‐editing for in vivo tumor treatment. This CRISPR/Cas12a nanotechnology for treating GBM through synergistic gene editing showed encouraging anti‐tumor efficacy that may be suitable for further translation study.

## 4 Experimental Section

### Materials

*N, N*ʹ‐bis(acryloyl)cystamine (BCA) and D‐Luciferin potassium salt were purchased from J&K Scientific (Beijing, China), Ammonium persulphate (APS, BioXtra, ≥98.0%, *M*_w_ = 228.2, Sigma‐Aldrich), *N, N, N', N'*‐Tetramethylethylenediamine (TEMED, ∼99%, *M*_w_ = 116.2, Sigma‐Aldrich), acrylate‐poly(ethylene glycol)‐*N*‐hydroxylsuccinimide ester (acryl‐PEG‐NHS, *Mn* = 2000 Da) were purchased from Jenkem Technology (Beijing, China). Acrylate guanidine was synthesized following the previously reported protocol.^[^ ^3b^ ^]^ ApoE peptides (LRKLRKRLLLRKLRKRLL) were obtained from China Peptide Co., Ltd (Suzhou, China). Cell culture medium, antibiotics (Penicillin and Streptomycin), and fetal bovine serum (FBS) were purchased from Gibco, Thermo‐Fisher. ApoE‐PEG‐acryl was synthesized by reacting between acryl‐PEG‐NHS and ApoE‐NH_2_. All other chemicals were obtained from Sigma‐Aldrich and utilized directly. Protein Cas12a was purchased from Integrated DNA Technologies, Inc. (Coralville, Iowa, USA). crRNA's template and primers were synthesized by Sangon Biotech Co., Ltd. (Shanghai, China). The sequences of the crRNA targeting human *EGFR* and *PLK1* are listed in Table S2 (Supporting Information).

### Preparation and Characterization of ANC@RNP Nanocapsules

Purified Cas12a protein (16.5 µg) was gently mixed with crRNA (3.2 µg) or pre‐crRNA (6.4 µg) at a 1:1.2 molar ratio within a 500 µL solution of 10 mm HEPES buffer (pH 7.4) in RNase‐free water at room temperature for 10–30 min. Acrylate guanidine, BCA, ApoE‐PEG‐acryl, APS and TEMED were accurately weighed and dissolved. The Cas12a RNP complex was stirred under nitrogen on ice. Then, 5 µg of acrylate guanidine (1 mg mL^−1^) and 66 µg of ApoE‐PEG‐acryl were added to the Cas12a RNP solution. After 5 min, 5.75 µg of BCA cross‐linker was introduced, along with 5 µL of APS catalyst (1 mg mL^−1^). The mixture was degassed for 5 min, and the polymerization reaction began immediately by adding 5 µL of TEMED under a nitrogen atmosphere. The polymerization proceeds for 2 h within a nitrogen atmosphere. Finally, any unreacted monomers and initiators were removed by dialysis in a 10 mm HEPES buffer (pH 7.4). The preferred formulation maintains a 1/220/220/220 molar ratio for Cas12a RNP/acrylate guanidine/ApoE‐PEG‐acryl/BCA. The Cas12a RNP/APS/TMEDA molar ratio remains consistent at 1/1/1. To create non‐targeted control nanoparticles, methoxy polyethylene glycol acrylate (mPEG‐acryl) was substituted for ApoE‐PEG‐acryl, while all other conditions remained the same. Transmission electron microscopy (TEM, JEM‐F200, JEOL) images and dynamic light scattering (DLS, Zetasizer Nano ZS, Malvern) were conducted to characterize the nanoparticles. For stabilities in long‐term storage, DLS were performed after various time's and temperatures’ incubation.

### In Vitro Transcription for crRNA

AsCas12a crRNAs and pre‐crRNA were synthesized by in vitro transcription using HiScribe T7 High Yield RNA Synthesis Kit (New England Biolabs) as described previously.^[^ ^36^ ^]^ The sequence of DNA oligonucleotides for the crRNA template are listed in Tables S3 and S4 (Supporting Information). Briefly, the annealed DNA oligonucleotides were utilized as templates for in vitro transcription reactions. T7 in vitro transcription was performed overnight, and then RNA was purified using RNA extraction reagent Trizol (Thermo‐Fisher). In vitrovalidation of crRNA was performed through in vitro cleavage assays using AsCas12a proteins. Briefly, AsCas12a protein (300 ng) and crRNA (100 ng) were pre‐mixed in 1x NEB2.1 buffer at room temperature for 5 min to pre‐assemble RNP complexes, which were then applied to cleave the crRNA genome target PCR amplicon (100 ng) at 37 °C for 1 h. The resulting PCR products were treated with RNase A and Proteinase K at 37 °C water‐bath for 30 min to degrade crRNAs and AsCas12a protein. The reaction samples were run on 2% Agarose gel stained with GelRed DNA stain, followed by imaging with a UV imager (Amersham Imager 680, GE).

### Cell Culture and Assessment of ANC@RNP Cellular Uptake by CLSM

U87MG cells were obtained from Shanghai Model organisms and maintained in Dulbecco's modified Eagle's Medium (DMEM) supplemented with 10% FBS (HyClone) at 37 °C with 5% CO_2_ incubation. Glioma stem cells (GSCs, X01 cells) were maintained in DMEM/F‐12 supplemented with B27 (Invitrogen, Thermo‐Fisher), EGF (10 ng mL^−1^, Invitrogen, Thermo‐Fisher), and bFGF (5 ng mL^−1^, Invitrogen, Thermo‐Fisher). A confocal fluorescent microscope was used to compare the intracellular distribution of ANC@RNP. U87MG cells were seeded on glass‐bottom dishes containing complete DMEM medium and incubated for 6 h. The final concentrations of ANC@RNP in the culture medium were 30 nm (for AF647‐Cas12a). To monitor cell morphology by visualizing the cell cytoskeleton, U87MG cells were stained with Alexa‐Fluor 488‐conjugated phalloidin (10 nm, Thermo‐Fisher) for 5–10 min in a live‐cell model. Subsequently, the cells were rinsed with PBS −0.1% Tween 20 three times (each for 5 min, 37 °C) and fixed with 4% formaldehyde for 15 min at room temperature. After another three rinses with cold PBS, the cell nuclei were stained with Hoechst 33 342 (5 mg mL^−1^) for an additional 3 min at 37 °C. Excess Hoechst was washed using cold PBS. Then, U87MG cells were imaged using a confocal laser scanning microscope (Zeiss 880, Germany). AF488, AF647, and Hoechst were excited using 488, 633, and 345 nm lasers, respectively.

### T7 Endonuclease I Assay Analysis for Gene Editing

Cells were incubated with various nanocapsules at 37 °C for 48 or 72 h before genomic DNA extraction. Genomic DNA was extracted using the Quick Extract DNA Extraction kit (Cwbio, Beijing) following the manufacturer's protocol. The genomic DNA fragment flanking the crRNA targeted sites was PCR amplified (primers listed in Table S5, Supporting Information), and PCR products were purified using a DNA Clean‐up Purification Kit (Cwbio, Beijing). 200 ng total of the purified PCR products were mixed with NEB buffer 2 and subjected to a re‐annealing process to enable heteroduplex formation: 95 °C for 10 min, 95 °C to 85 °C ramping at −2 °C s^−1^, 85 °C to 25 °C at −0.25 °C s^−1^, and 25 °C hold for 1 min. After re‐annealing, PCR products were digested with 10 units of T7E1 (NEB, #M0302) at 37 °C for 30 min and run on 2% Agarose gels followed by imaging with an imaging system (Amersham Imager 680, GE). Indel percentage was determined by the formula, 100 × (1 − (1 − (b + c)/(a + b + c))^1/2^), where a is the integrated intensity of the undigested PCR product and b and c are the integrated intensities of each cleavage product.

### Uptake and Apoptosis Induction of ANC@RNP Determined by Flow Cytometry

For cell uptake, U87MG cells were seeded onto a 12‐well plate at a density of 4 × 10^4^/well overnight, followed by incubation for 6 h with AF647 labeled ANC@RNP. The final concentration of RNP in the culture media was 30 nm. Then, the U87MG cells were digested with trypsin and washed with PBS. The AF647 signals were collected with CytoFlex LX, Beckman. For apoptosis, U87MG cells were seeded onto a 6‐well plate at a density of 1.2 × 10^5^/well overnight before incubation with various nanocapsules that carried Cas12a/crRNA. Then, 96 h after incubation, U87MG cells were double‐stained with annexin V and propidium iodide using the Annexin V‐PI apoptosis detection kit (Vazyme Biotech) according to the manufacturer's instructions. The flow data were collected using CytoFlex LX, Beckman at excitation wavelengths of 638 nm and analyzed using FlowJo v10.

### RNA Sequencing

RNA‐seq experiments were conducted by Shanghai Biotechnology. Briefly, U87MG cells were collected using RNA extraction reagent Trizol (Thermo‐Fisher) after 96 h incubation with various nanocapusles@RNP. The first and second‐strand cDNA synthesis and libraries construction were performed according to the manufacturer's instructions. The cDNA then underwent enzymatic fragmentation, end repair, and size selection to optimize its size. Then, adaptor ligation, sample index PCR, and sided size selection were performed to generate the cDNA libraries. The libraries were quality‐controlled with Agilent Technologies 2100 Bioanalyzer. Libraries were sequenced on the Illumina Hiseq 2500 platform. The top 200 DEGs for the cell population of interest were selected for Gene Ontology (GO) and Kyoto Encyclopedia of Genes and Genomes (KEGG) enrichment analysis using NCBI GO/KEGG database.

### Penetration and Assessment in U87MG 3D‐Spheroid Tumor Model

The U87MG 3D tumor spheroids were established as previously reported.^[^ ^21^ ^]^ Briefly, U87MG cells (4 × 10^3^ per well) were plated in low attachment 96‐well plates (Sumitomo Bakelite, Japan). After 3 days, the 3D tumor spheroids were treated with various AF647 labeled ANC@RNP for another 4 h incubation. Then, tumor spheroids were washed with PBS plus 0.05% Tween 20 and fixed with 4% paraformaldehyde. The permeability of samples into tumor spheroids was assessed by Confocal (Zeiss 880, 10×magnification).

### Western Blotting

For Western Blotting, U87MG cells and mice glioma tissue was lysed in RIPA in the presence of a protease inhibitor (PMSF) and quantified using a BCA assay. The proteins were separated using SDS‐polyacrylamide gel electrophoresis (PAGE) and transferred to a polyvinylidene difluoride membrane (Millipore). The membranes were blocked by 5% BSA dissolved in TBS/0.1% Tween‐20 for 2 h and incubated with monoclonal antibodies overnight at 4 °C (anti‐PLK1, EGFR, p‐STAT3, p‐AKT, p‐ERK, c‐Myc, 1:1000, Abcam). The membranes were incubated with HRP‐conjugated anti‐mouse secondary antibodies (1:3000, Cell Signaling Technology) and visualized using chemiluminescence (Amersham Imager 680, GE). GAPDH was detected using an anti‐GAPDH monoclonal antibody (1:3000, Cell Signaling Technology) for internal control across samples.

### GBM Xenograft Mouse Model Preparation and In Vivo Bioluminescence Imaging

GSCs‐luc cells were kindly provided by Jong Bae Park lab at National Cancer Center, Goyang, Republic of Korea. GSCs‐luc cells were maintained in DMEM/F12 supplemented with EGF (10 ng ml^−1^; R&D Systems), bFGF (5 ng ml^−1^; R&D Systems), B27 (ThermoFisher), and 1% Pen/Strep (ThermoFisher). To prepare the U87MG and GSC xenograft mouse models, ≈4 × 10^6^ cells in 100 µl of culture media mixed with 100 µl of matrigel (BD Biosciences) were implanted orthotopically in the brain of 6–8 weeks male nude mice as described previously.^[^ ^21^ ^]^ Briefly, the 8–12 weeks of male BALB/c nude mice were purchased from SiPeiFu (SPF) (Beijing) Biotechnology Co., Ltd. All animal experimental work was performed with the approval of the Institutional Animal Care and Use Committee, Henan University Health Science Center. Before conducting all the surgical injection procedures, the animals were anesthetized. Mice were monitored for GBM growth every second day. Assessment of anti‐tumor efficacy xenograft tumor model was first performed by anesthetizing the mice with isoflurane. Mice were then positioned prostrate. D‐luciferin (Xenogen) in PBS was used as a substrate to mediate the monitoring of tumor progression after intraperitoneal injection (15 mg kg^−1^). Bioluminescence images were collected with an IVIS Spectrum in vivo Imaging System (PerkinElmer). Acquisition times ranged from 10 s to 5 min.

### In Vivo Pharmacokinetics and Distribution Study

For the in vivo pharmacokinetics study, a healthy BALB/c male mouse (6–8 weeks) was used. The mice were randomly divided into three groups (*n* = 3 per group) and intravenously administered with the following preparations (free Cas12a RNP, NC@RNP, ANC@RNP) at an AF647‐Cas12aRNP dose of 1.5 mg per kg of animal weight. Blood was collected from the retro‐orbital vein with a heparin‐coated capillary tube at predefined time intervals (0, 5, 15, 30, 60, 120, 180, and 240 min), followed by brief centrifugation into a 1.5 mL tube. The fluorescence intensity of AF647 was measured at excitation and emission wavelengths of 640 and 670 nm, respectively, using a microplate reader (SpectraMax i3x, Molecular Devices). The pharmacokinetics curve was created by estimating the percentage of AF647‐Cas12a in blood at each time point and normalizing it to the starting (0 min) time point. For the biodistribution study, U87MG xenograft‐bearing male nude mice received the following preparations (free Cas12a RNP, NC@RNP, ANC@RNP) through the tail vein at a dose of 1.5 mg Cas12a RNP per kg of mice weight (*n* = 3 per group). Live images were taken with an IVIS Imaging System (Perkin Elmer) at various predefined time intervals within 24 h. Main organs and brains were collected 8 h after injection. For in vivo biodistribution, AF647 fluorescence was quantified for each specific tissue/organ using the IVIS Spectrum imaging system. For quantification analysis, the main organs and tumor tissues were homogenized and measured using a microplate reader, and the percentage of injected dose per gram of tissue (%ID/g) was calculated. Brain tissues were sectioned and scanned with a confocal image to examine the distribution of AF647 delivered by nanocapsules in the brain tissue.

### In Vivo Therapeutic Efficacy of ANC@RNP

To assess the in vivo therapeutic efficacy of the Cas12a RNP, two different mice models of GBM, U87MG‐Luc, and GSC‐Luc‐derived orthotopic xenografts were prepared, respectively. Mice were monitored for GBM growth every 2 days using an IVIS imaging bioluminescence system after mice were injected intraperitoneally with luciferin substrate (15 mg kg^−1^). At 9‐ or 10‐days post‐implantation (DPI), mice were randomly divided into six treatment groups (*n* = 5 per group). Anti‐GBM treatments were administered via *i.v*. injection at a dose of 1.5 mg RNP per kg of mice weight for a total of five doses. GBM bioluminescence images were monitored every 2 days from the day of initial treatment (9 or 10 DPI) to monitor the anti‐GBM effect using the IVIS imaging system.

### Histology and Immunohistochemistry

Mouse brains were removed after with ANC@RNP or control treatments, fixed with 4% paraformaldehyde (PFA) for 24 h at 4 °C, sectioned at a thickness of 5 µm using an essential microtome (Leica, RTS), and stained with hematoxylin and 0.25% eosin to monitor glioma progression. Additionally, major organs were collected, sectioned, and H&E stained for in vivo safety investigations. For immunohistochemical staining of apoptosis and tumor proliferation markers, brain‐containing tumor sections were subjected to an antigen retrieval process using citrate buffer (pH 6.0), and endogenous peroxidases were blocked by incubating with 3–5% hydrogen peroxide (H_2_O_2_), followed by washing with ddH_2_O. Brain sections were then incubated overnight at 4 °C in a humidified chamber with primary antibodies (CCK3, Ki67, 1:500 diluted with diluent buffer). Tumor sections for DAB staining used 3,3′‐diaminobenzidine (DAB; Vector Laboratories) as the chromogen. Apoptosis of tumor tissue was determined by the terminal deoxynucleotide transferase (TdT)‐mediated dUTP nick‐end labeling (TUNEL) assay using an in situ cell death detection kit (KeyGEN, Nanjing, China) following the manufacturer's protocol. For immunofluorescence staining, slides with brain sections were warmed to 37 °C in an incubator; then, sections were marked with a hydrophobic marker (blue) and dried on a warmer (37 °C), followed by rehydration with PBS. Then, immunofluorescence staining was performed in a moist, dark incubation chamber. Sections were permeabilized with TBS/0.1% Triton‐X100 and blocked with TBS‐5% donkey serum. After DAPI nuclear staining, sections were washed in TBST and covered with coverslips under a mounting reagent (Prolong Diamond, Thermo‐fisher). The sections were imaged by confocal microscopy (Zeiss 880, Germany) and analyzed using ZEN software.

### In Vivo Toxicity Evaluation

To evaluate the in vivo toxicity, blood was collected from healthy BALB/c mice at several time points after a single injection of ANC@RNP or control treatments. The liver function (AST, ALT, and ALB) and renal function (BUN, CR, and UA) were determined by Wuhan Servicebio Technology Co. At 2‐ or 14 h post‐injection, serum samples were collected and processed to measure the representative cytokine (TNF‐α) by qRT‐PCR according to the manufacturer's protocol. For qRT‐PCR, RNA was extracted from blood with the kit (RNApure Blood Kit, CWbio, Beijing), followed by quantification using a Nanodrop3000 (Thermofisher) and stored at −80 °C. RNA was reverse transcribed using PrimeScript RT Master Mix (Takara) according to the manufacturer's instructions. qPCR was performed in triplicate on LigheCycler480 (Roche) using a One‐step PCR Mastermix (Takara). GAPDH was used as a housekeeping gene. The primers are listed in Table S6 (Supporting Information).

### Targeted Deep Sequencing of Candidate Off‐Target Sites

Target and potential off‐target sites were analyzed by targeted deep sequencing using Illumina. To evaluate off‐target effects, the web‐based tool Cas‐OFFinder (http://www.rgenome.net/cas‐offinder) was used with the hg38 reference genome to predict the putative off‐target site listed in Table S7 (Supporting Information). Deep sequencing libraries were generated by High Fidelity PCR with 20 ng gDNA template. PCR was performed as follows: 4 min at 98 °C for initial denaturation, followed by 28 cycles of 15 s at 98 °C for denaturation, 15 s at 60 °C for annealing, 30 s at 72 °C for extension, and 7 min for the prolonged extension. The PCR amplicons were purified using a DNA Clean‐up Purification Kit (Cwbio, Beijing), followed by size validation with 2% agarose electrophoresis. The PCR amplicons were then assessed for gene editing efficiency using next‐generation deep sequencing (NGS) by GENEWIZ Company (Suzhou, China).

### Statistics

All statistics data were presented as means ± standard error of the mean (SEM) from>3 independent experiments. Kaplan‐Meier analysis was used to plot survival curves. The statistical software GraphPad 8 was used for statistical analysis. The results of two‐dataset experiments were compared using a two‐tailed Student's t‐test. Results were considered statistically significant at p‐values of *p* < 0.05, with *p* < 0.01 and *p* < 0.001 considered statistically very significant.

## Conflict of Interest

The authors declare no conflict of interest.

## Supporting information

Supporting Information

## Data Availability

The data that support the findings of this study are available from the corresponding author upon reasonable request.
