# Supplementary material for: Brain‐Targeted Cas12a Ribonucleoprotein Nanocapsules Enable Synergetic Gene Co‐Editing Leading to Potent Inhibition of Orthotopic Glioblastoma
Source: Adv Sci (Weinh). 2024 Jun 28;11(33):2402178. doi: 10.1002/advs.202402178 (PMC11434219; doi:10.1002/advs.202402178)
Supplement: Supplementary file 1 — Supporting Information [file ADVS-11-2402178-s001.docx]

Supplementary Materials for

**Brain-targeted Cas12a ribonucleoprotein nanocapsules enable** **synergetic gene co-editing leading to potent inhibition of orthotopic glioblastoma**

Weimin Ruan, Sen Xu, Yang An, Yingxue Cui, Yang Liu, Yibin Wang, Muhammad Ismail, Yong Liu*, Meng Zheng*

*Dr. W. Ruan, S. Xu, Y. Cui, Y. Liu, B. Wang, M. Ismail, M. Zheng*

*Henan-Macquarie University Joint Centre for Biomedical Innovation, Henan Key Laboratory of Brain Targeted Bio-nanomedicine, Henan International Joint Laboratory of Nanobiomedicine, School of Life Sciences, Henan University, Kaifeng, Henan, 475004, China*

Email: [mzheng@henu.edu.cn](mailto:mzheng@henu.edu.cn) (M. Zheng)

*Dr.Y, An*

*Henan Provincial Engineering Center for Tumor Molecular Medicine, School of Basic Medical Science, Henan University, Kaifeng 475004, China*

*Dr. Yong, Liu*

*Laboratory of Nanoscale Biosensing and Bioimaging, School of Ophthalmology and Optometry, School of Biomedical Engineering, Wenzhou Medical University, 270 Xuanyuanxi Road, Wenzhou, Zhejiang 325027, China*

Email: [yongliu@wmu.edu.cn](mailto:yongliu@wmu.edu.cn) (Y. Liu)

W. Ruan and S. Xu contributed equally to this work.

Figure S1. Selection and validation of effective crRNA of EGFR and PLK1. Cas12a RNP was transfected with lipoCRISPRmax and incubated with U87MG cells at a dose of 30 nM Cas12a. Genome DNA was isolated after 48 h transfection, followed by T7E1 assay detection. The arrows indicate the cut DNA band.


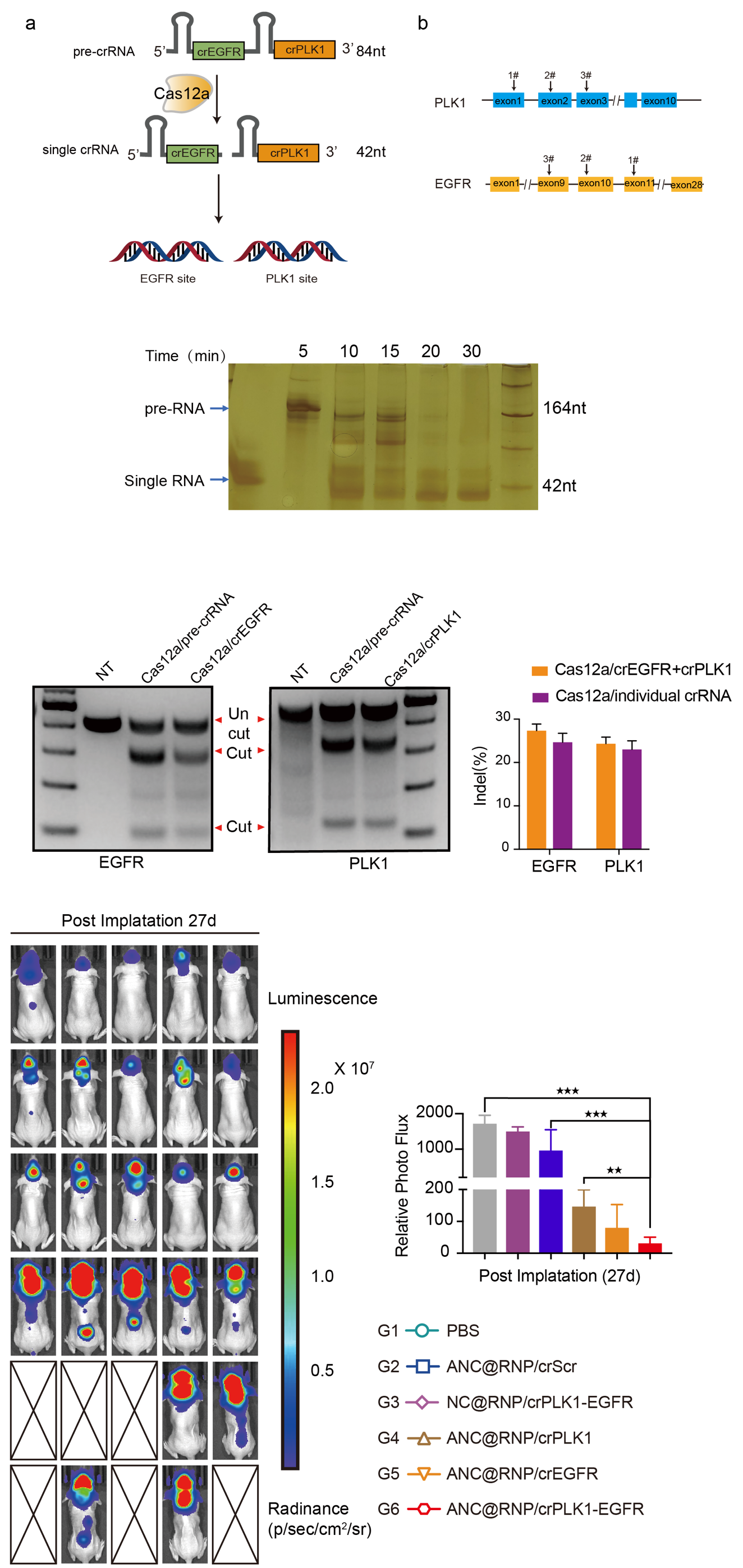


Figure S2. Cas12a-mediated pre-crRNA processing *in vitro* is incubation-time dependent. The Cas12a to pre-crRNA ratio was 1:1 (molar ratio). Incubation time was extended to 20 min to allow for complete pre-crRNA processing. Then, RNA in the incubation mixture was detected by de-natured 6M Urea -10% PAGE electrophoresis, followed by silver staining. The sequence of pre-crRNA is listed in Supplementary Table 4.


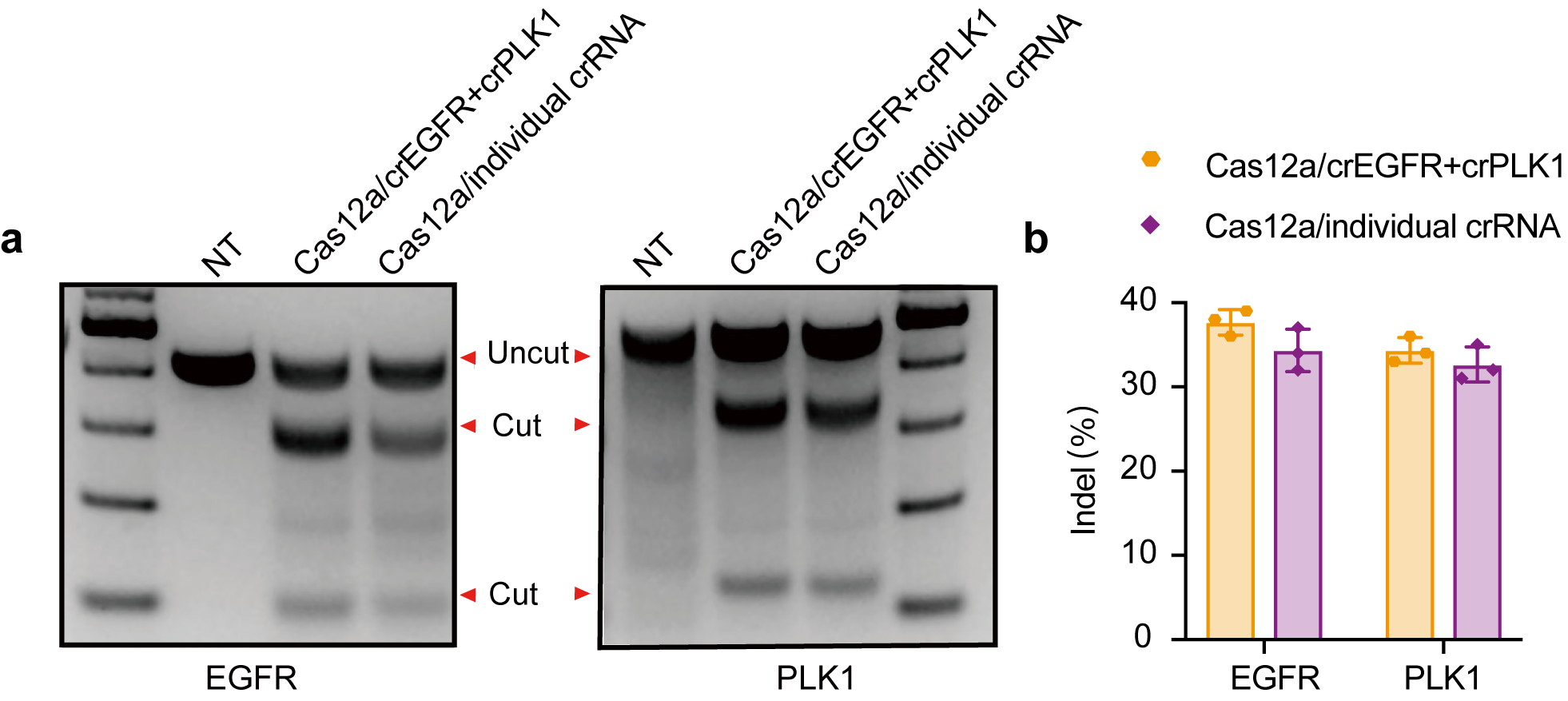


Figure S3. Indel rates of the *EGFR* and *PLK1* genes edited by Cas12a RNP. U87MG cells were transfected with LipoCRISPRmax and Cas12a RNP for 48 h with Cas12a protein (30 nM). T7E1, which could recognize and cleave mismatched PCR amplicons, was used to digest the heteroduplexes for indel analysis.

**
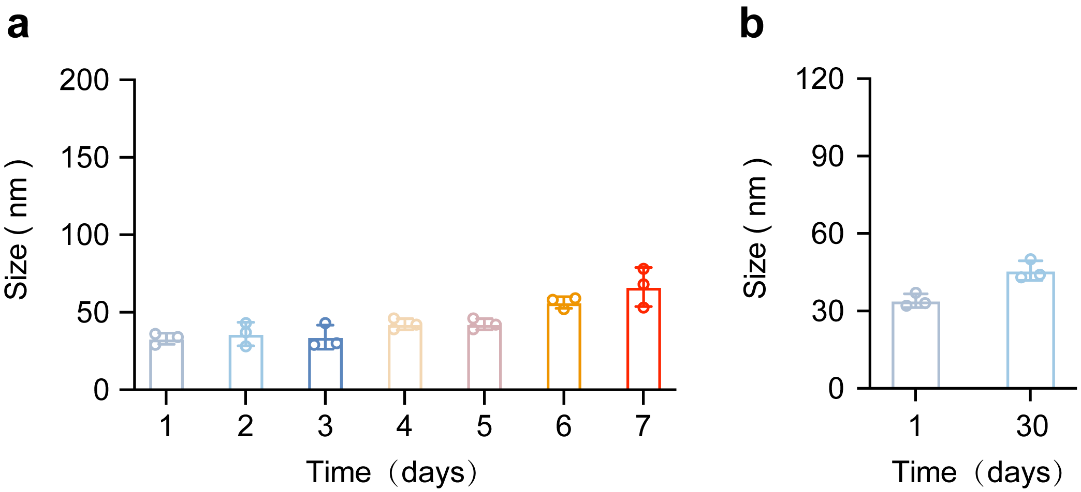
**

**Figure S4.** **Stabilities of ANC@RNP.** DLS was performed to indentify the ANC@RNP nanocapsules’ size in PBS buffer, under **(a)** physiological condition (37 °C) for 1 week and **(b)** 4°C storage for 1 month.


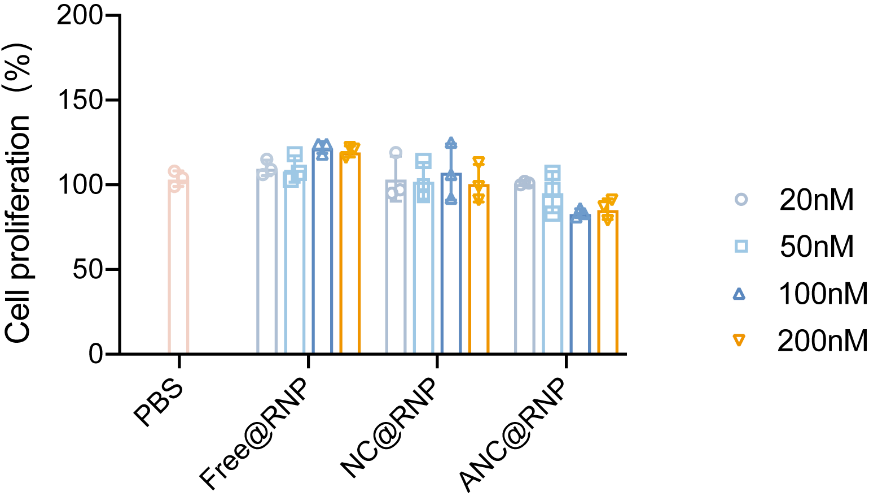


Figure S5. Cell viability of U87MG cells after 72 h incubation with ANC or controls. Data are normalized to cells incubated with PBS. Data are mean ± SD (n=3).


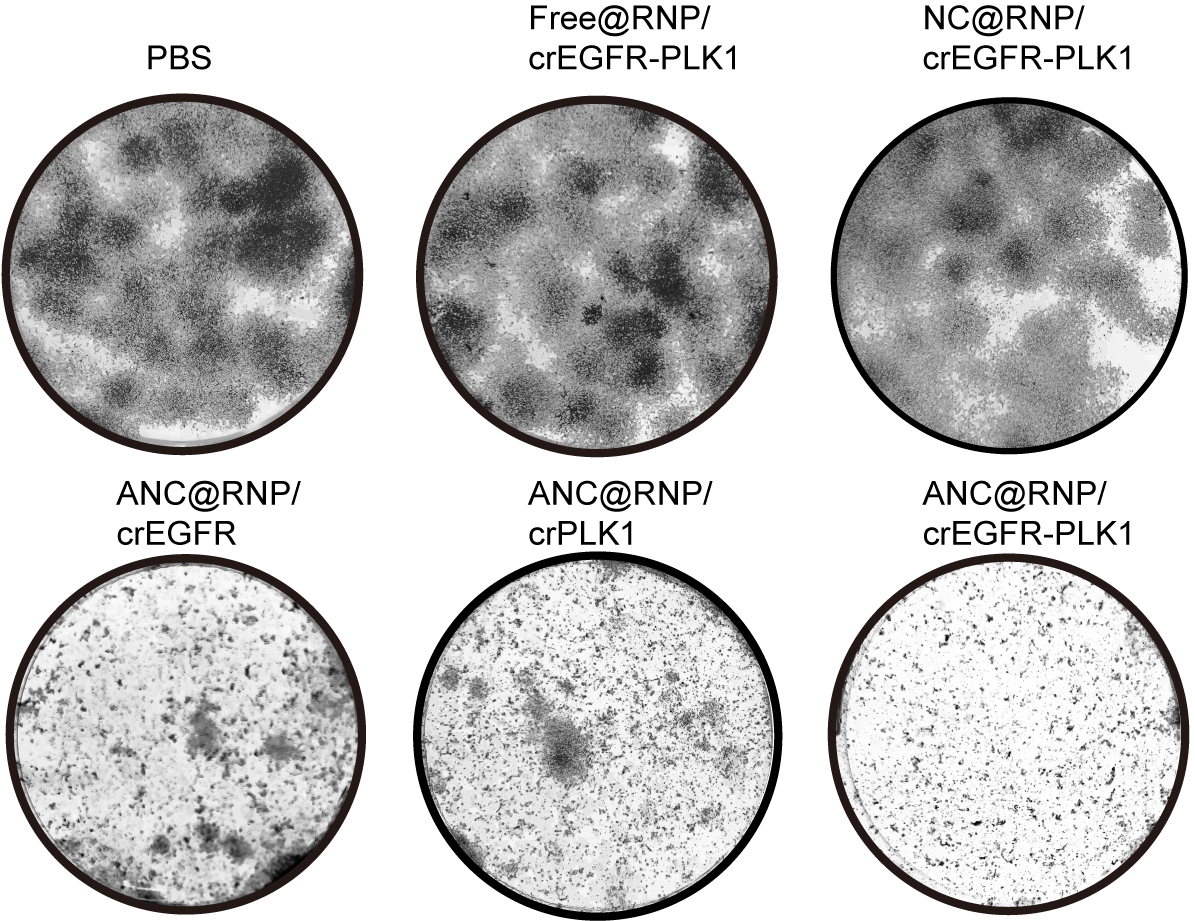


**Figure S6.** **Representative images of colony formation assays after treatment with ANC.** Colony formation assay showed that ANC@RNP/crEGFR-PLK1 inhibits U87MG glioma cells from forming colonies, exhibiting synergetic effects. Cas12a protein concentration was 30 nM.


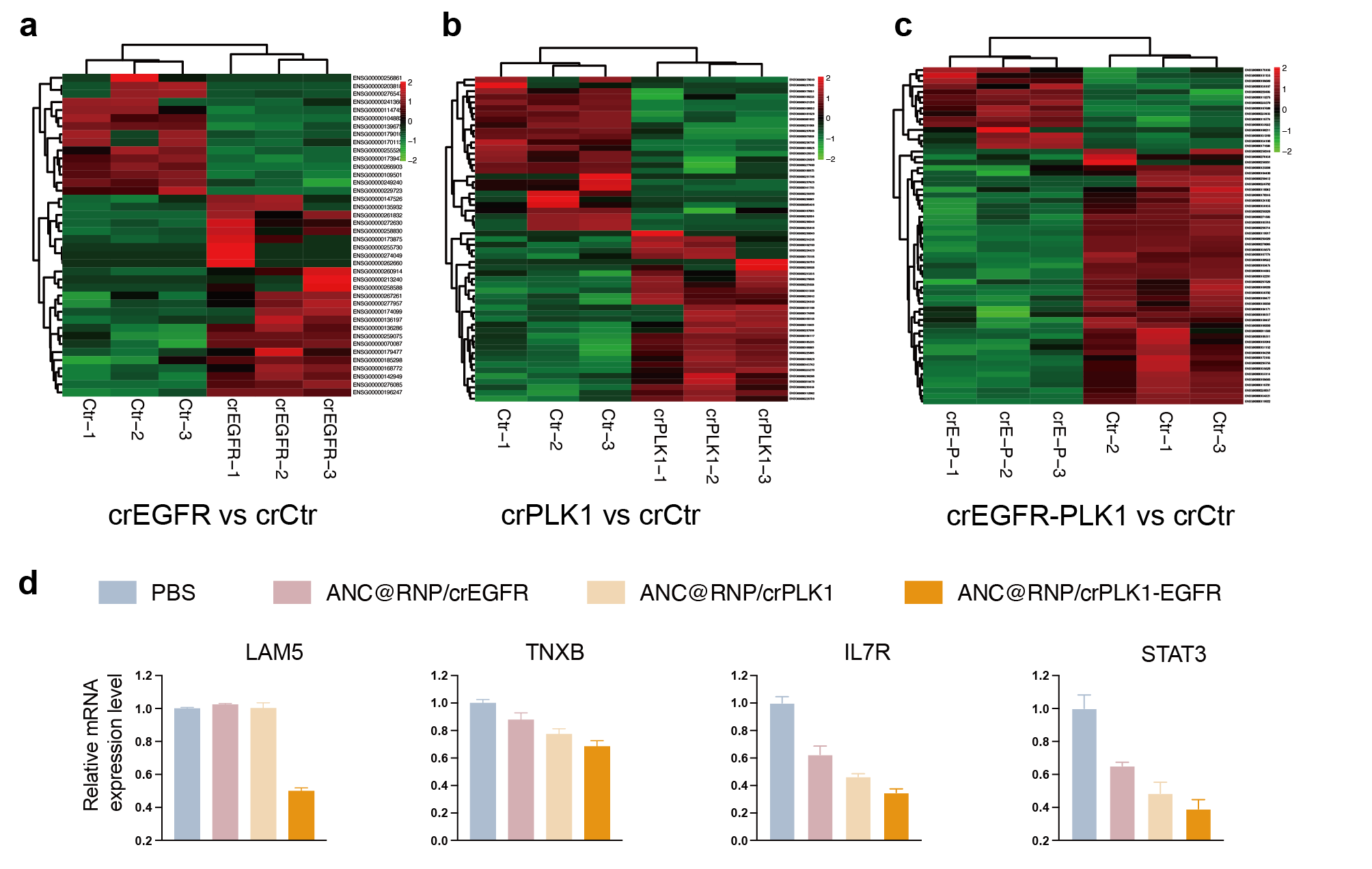


Figure S7. Heat map of RNA sequencing of U87 cells after 48 h treatment with ANC@crEGFR, ANC@crPLK1, ANC@crEGFR-PLK1 and ANC@crCtr. (a-c) Heatmap comparison of the gene expression profile of genes after treatment. (d) qPCR validation of the expression of PI3K-Art and JAK-STAT-related genes.

**
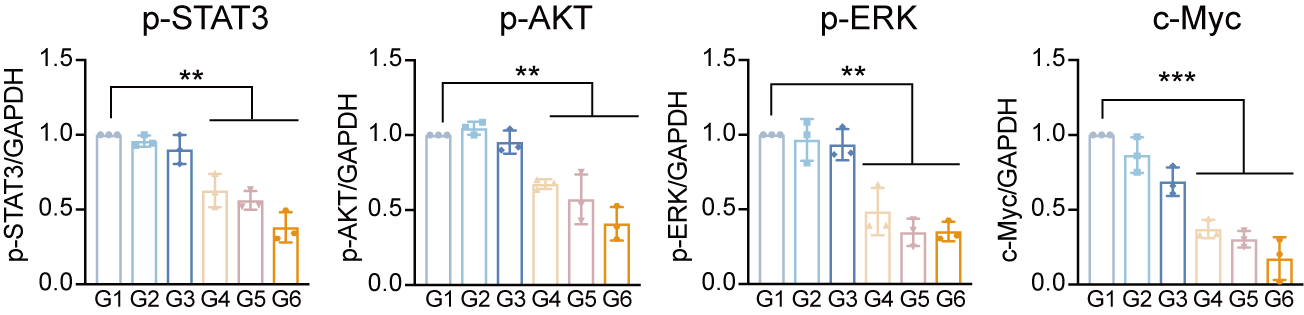
**

Figure S8. Quantitation of Western Blot data of downstream protein and c-Myc expression relative to GAPDH in Figure 2D. Data are mean ± SD ( n=3; student t-test; **p<0.01; ***p<0.001 ).


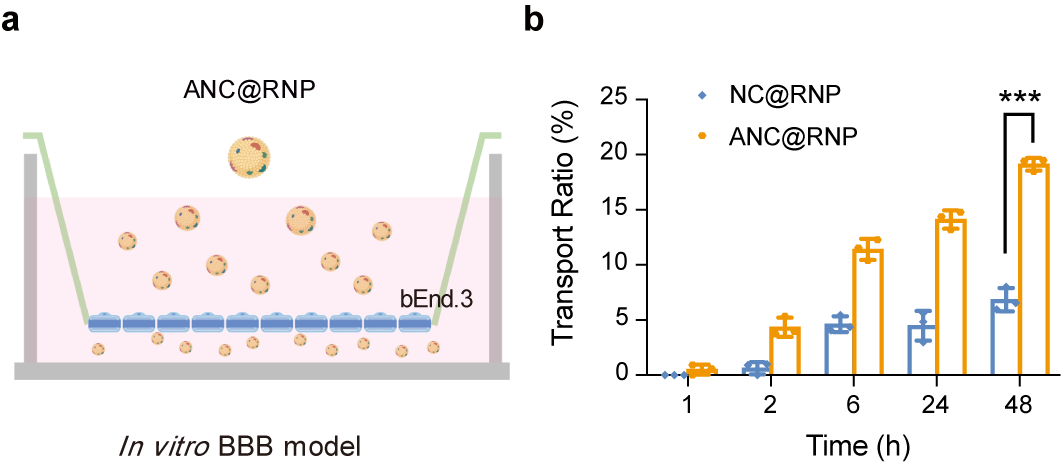


Figure S9. Transwell assay. (a), Schematic of BBB in vitro model seeded with bEnd.3 cells. (b), Cumulative transport ratio of ANC and NC nanocapsules across the *in vitro* BBB barrier at 1, 2, 6, 24, and 48 hours. Data are mean ± SD (n=3; student t-test; **p<0.01).


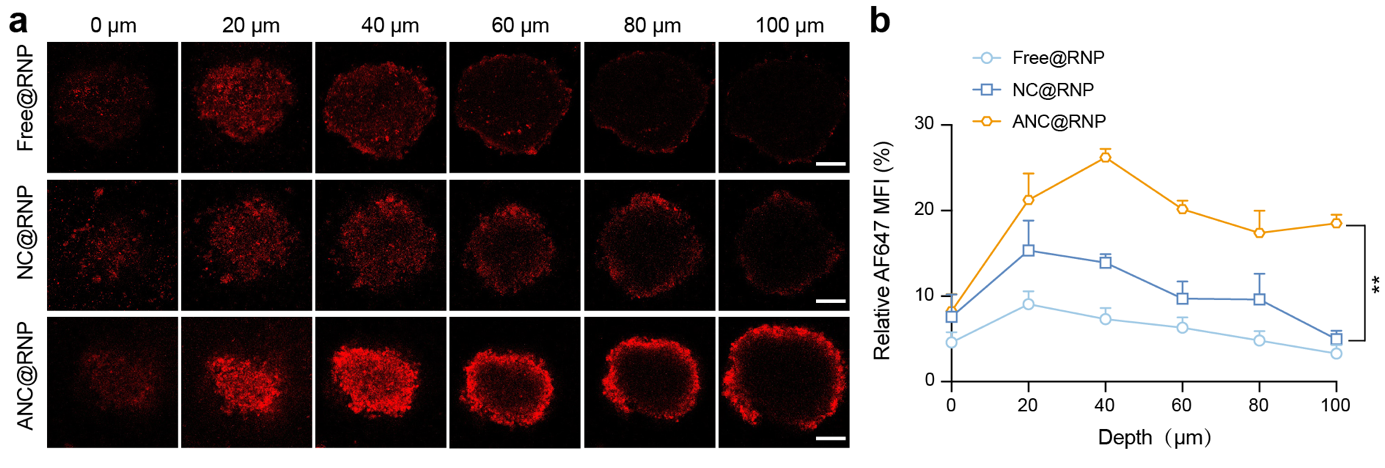


Figure S10. Penetration of ANC nanocapsules into U87MG 3D spheroids. (a), Penetration of ANC nanocapsules into U87MG 3D multicellular spheroids after 8 h incubation (AF647-Cas12a concentration was 30 nM). Z-stack images were captured starting from the top and progressing to the spheroid core at intervals of 20 µm. Scale bar = 20 μm. (b), Quantitative analysis of penetration assay in a. Data are mean ± SD (n=3, **p<0.01).


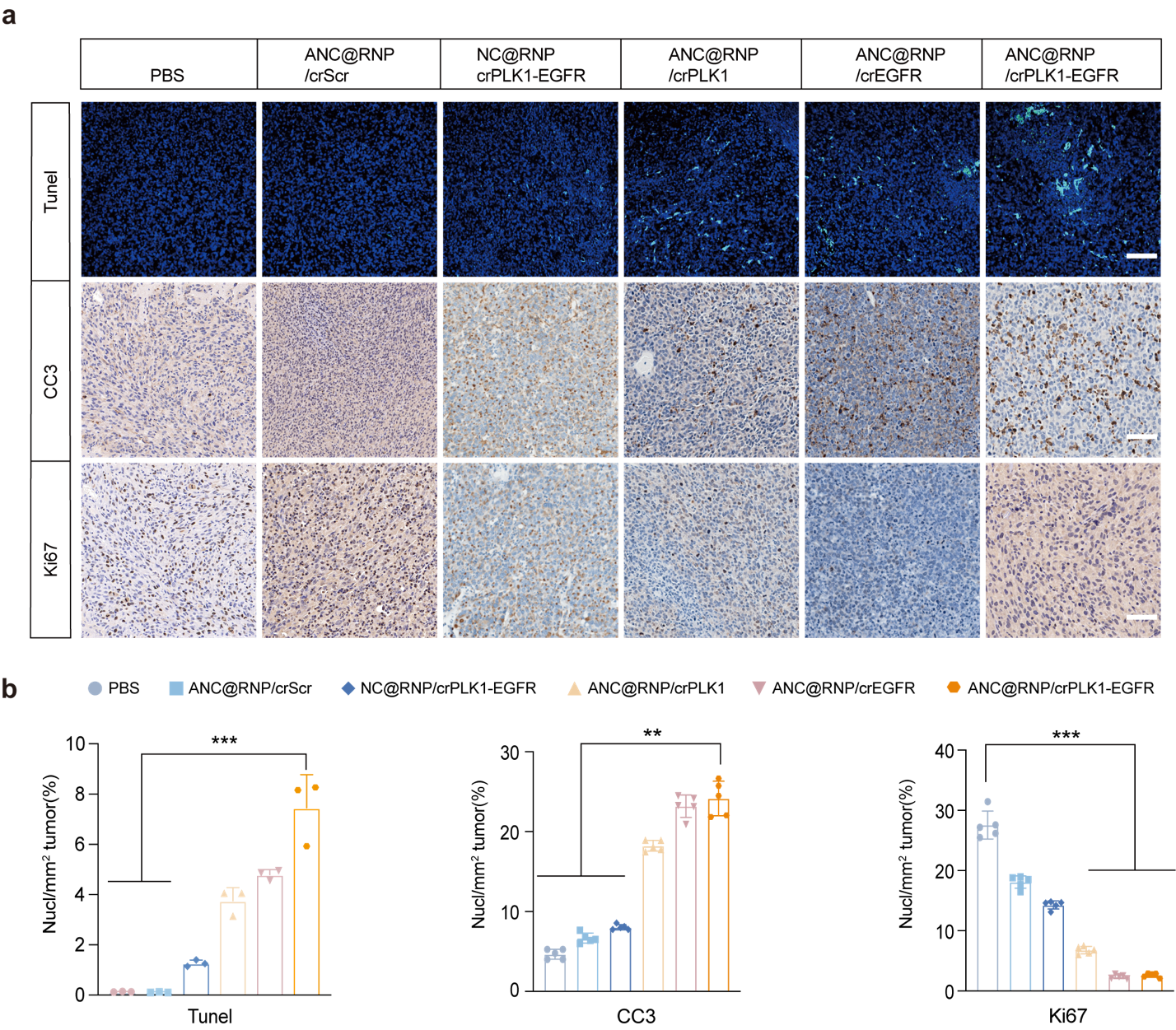


Figure S11. Apoptosis and proliferation markers in excised brain tumor tissues from U87 xenograft tumor model treated with various nanocapsules and controls. (a), Tumor slices excised from orthotopic U87MG-Luc human glioblastoma tumor-bearing nude mice following treatment with ANC@Cas12a/crEGFR-PLK1, ANC@Cas12a/crEGFR, ANC@Cas12a/crPLK1, NC@Cas12a/crEGFR-PLK1, ANC@Cas12a/crScr (1.5 mg Cas12 protein equiv./kg) or PBS and stained for TUNEL, cleaved caspase 3 (CC3) or the proliferation marker Ki- 67. (b), Quantifying the number of tumor cells staining positive for TUNEL, CC3 or Ki-67. Signal intensity was quantified from over 300 cells in tumors of mice using ImageJ software. Scale bars are 100 μm for TUNEL, CC3, and Ki-67, respectively. Data are mean ± SD (**p<0.01, ***p<0.001).


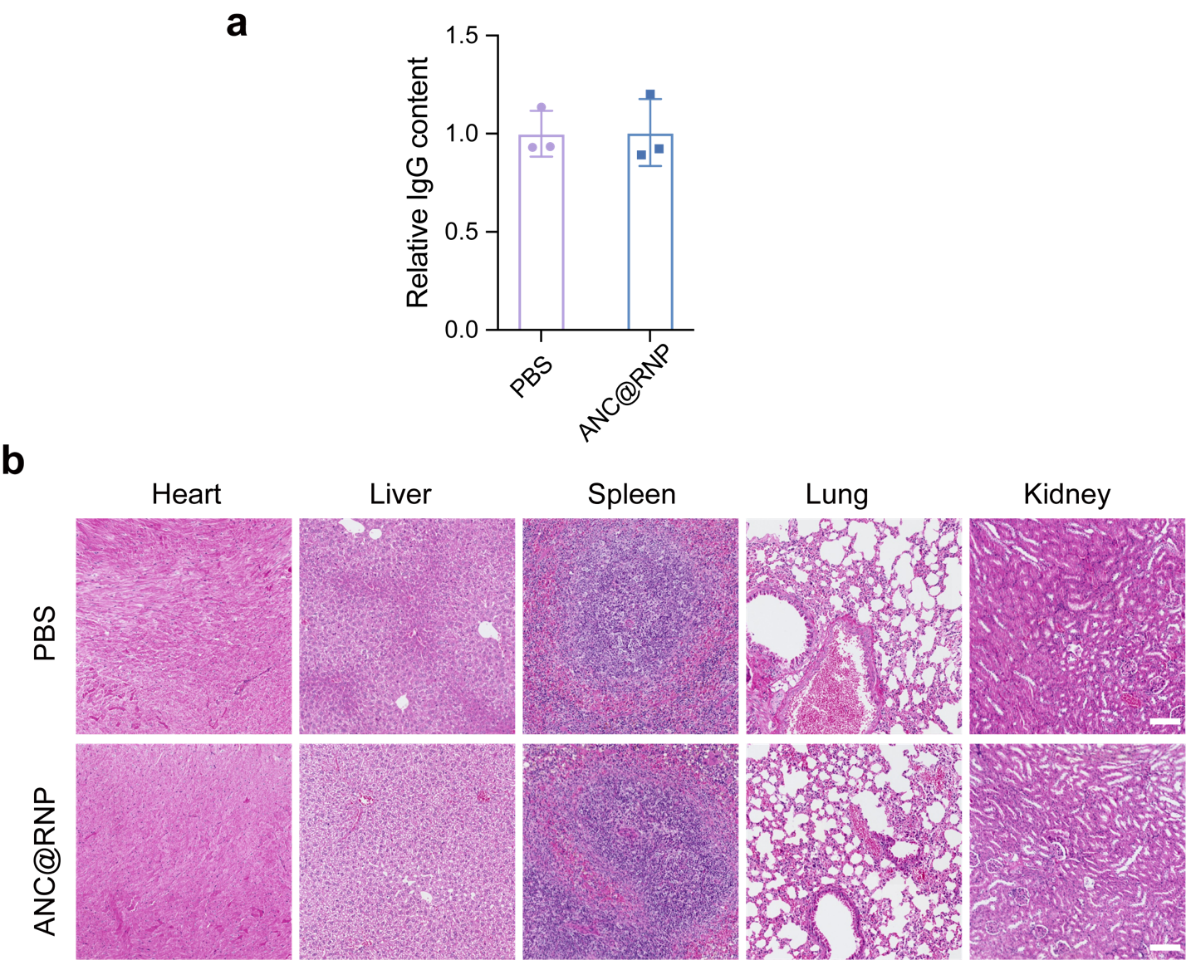


Figure S12. Immunogenicity and bio-safety of ANC@RNP in health mice. (a), IgG levels’ change in healthy mice blood on days 14 after single administration of ANC@RNP. Detected by Elisa assay. (b), Representative H&E staining images of major organs excised from healthy BALB/c mice. The major organs (heart, liver, spleen, lung and kidney) were excised from a healthy mouse after 14 days of receiving single administration of ANC@RNP, Scale bar: 50μm.


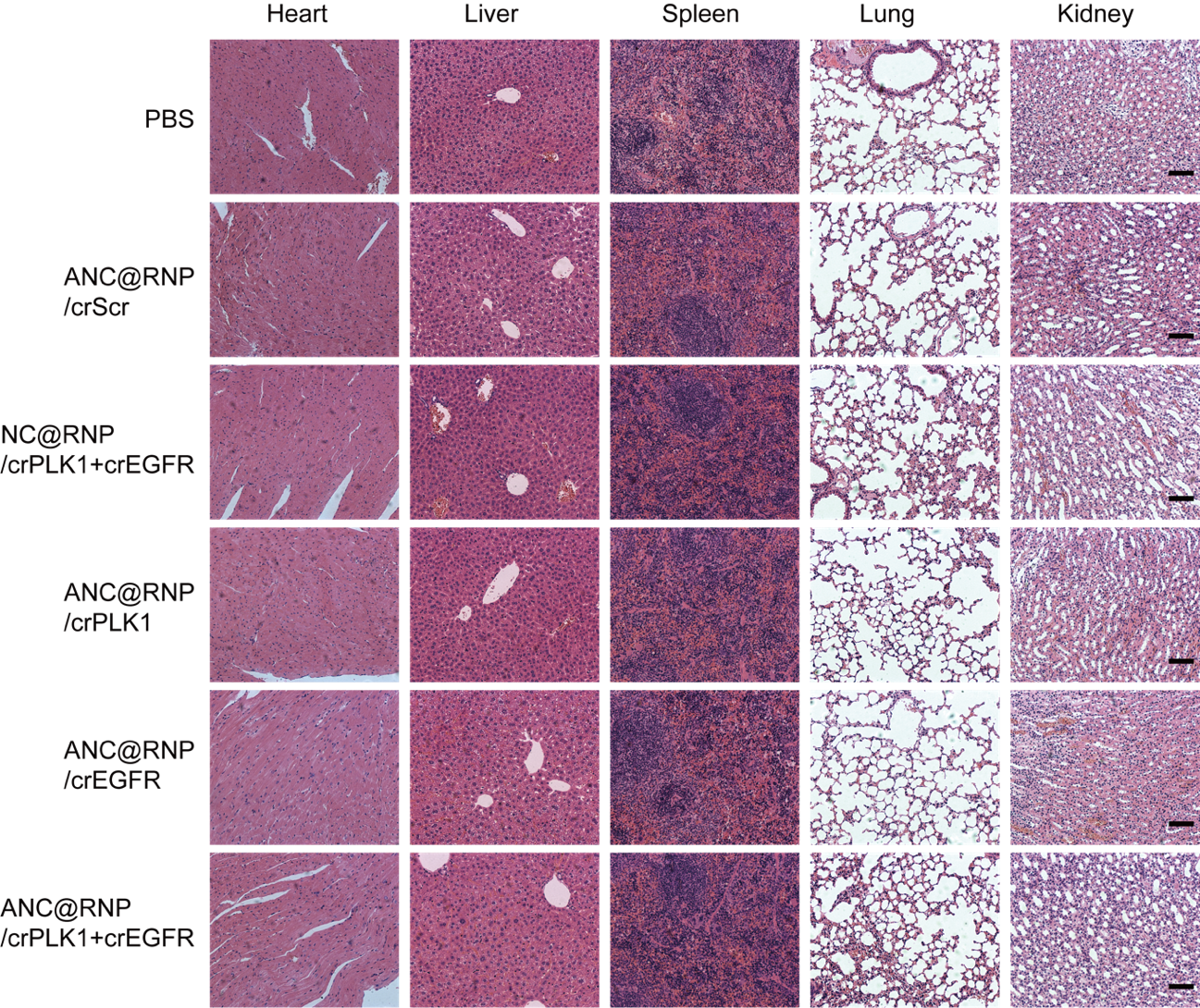


Figure S13. Representative H&E staining images of major organs excised from U87-bearing mice. The major organs (heart, liver, spleen, lung and kidney) were excised from a mouse receiving ANC@RNP/crEGFR-crPLK1, Scale bar: 50μm.


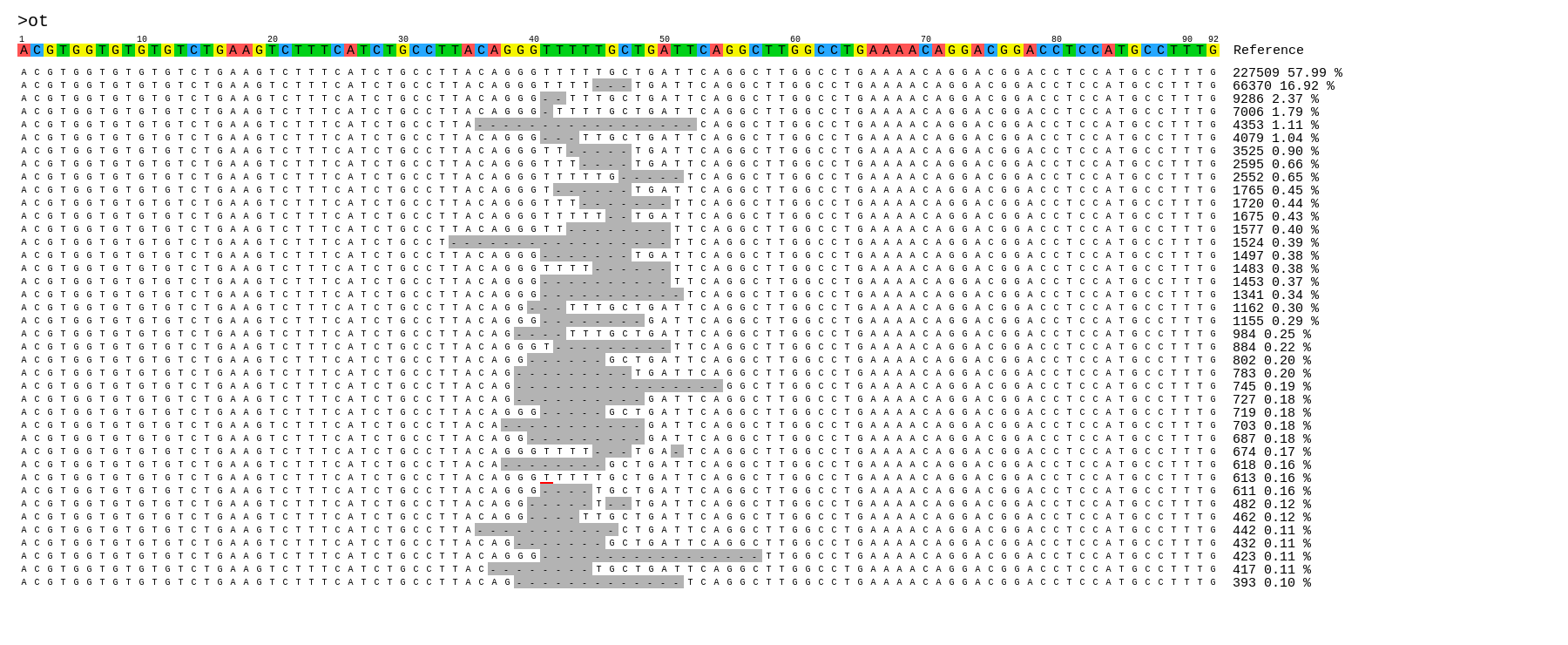


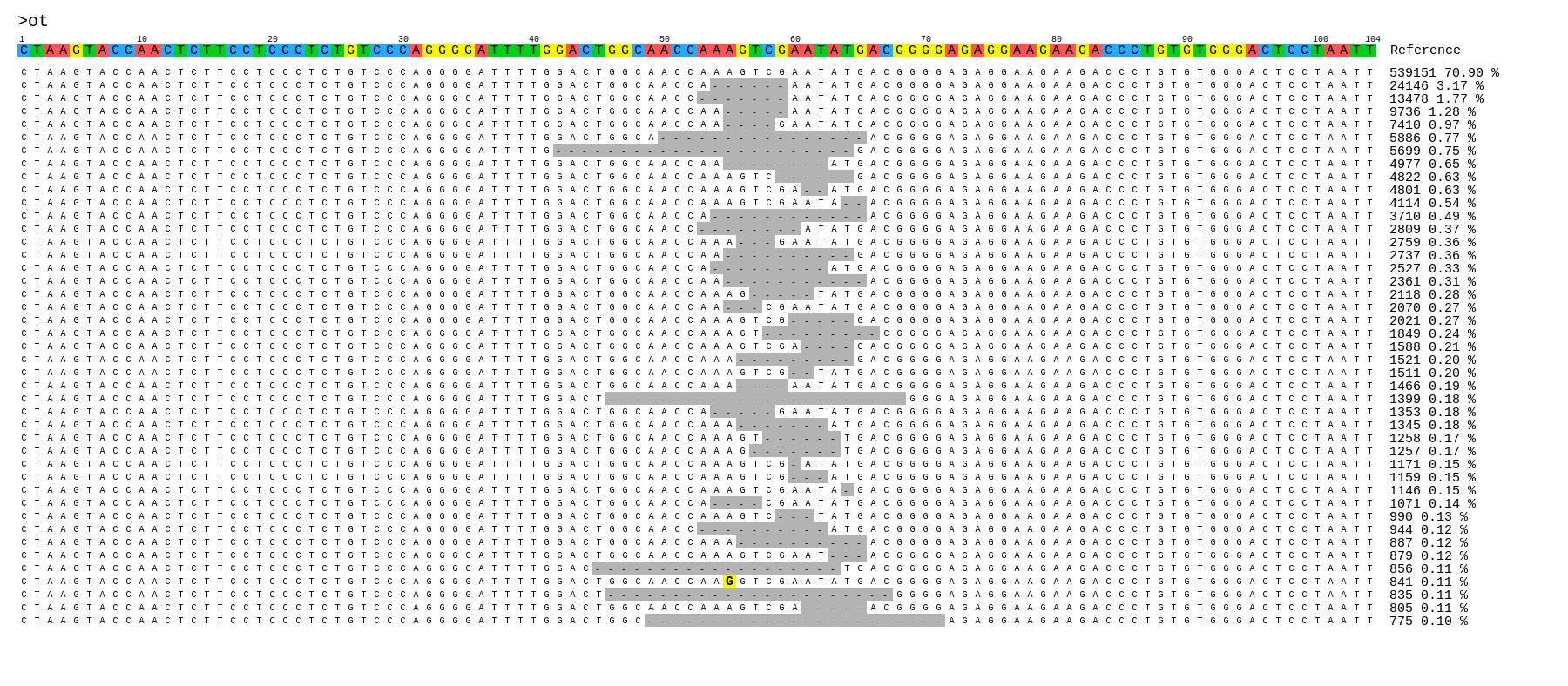


Figure S14. NGS sequencing confirms indels in the U87-model GBM tissue and Sanger sequencing. Up panel, EGFR site. Lower panel, PLK1 site. Deletion spectra are labelled in gray.


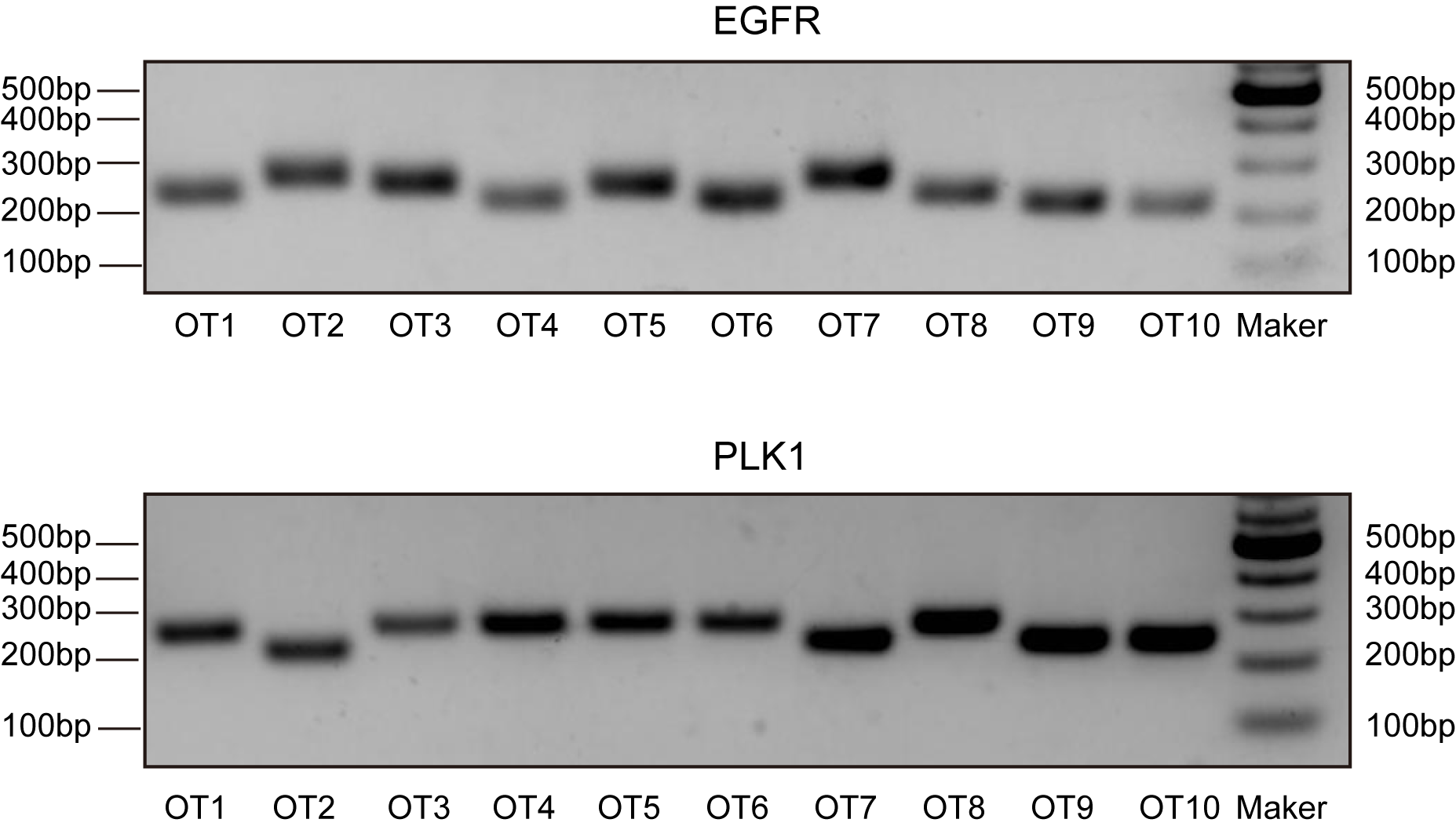


Figure S15. No off-target gene editing was detected by T7E1 assay in GSC model mice. GSC model mice were treated with ANC@ nanoparticles targeting gene EGFR and PLK1. The top 10 potential off-target of crEGFR and crPLK1 sites were amplified by PCR and analyzed by T7EI assay. No cleaved products were detected for all 20 potential off-target sites. The experiment was independently repeated three times with similar results.

Table S1. Size distribution and zeta potential of free Cas12a RNP determined by DLS.

| Particles | Size (nm) | PDI | Zeta potential (mV) |
| --- | --- | --- | --- |
| Free Cas12a RNP | 11±4 | 0.56 | -22 |
| NC@RNP/crEGFR-PLK1 | 29±3 | 0.27 | +9 |
| ANC@RNP/crEGFR | 31±3 | 0.21 | +7 |
| ANC@RNP/crPLK1 | 32±4 | 0.26 | +7 |
| ANC@RNP/crEGFR-PLK1 | 34±3 | 0.24 | +9 |

Table S2. Target sequences for crRNA in this study

| Target site | Target site (5'-3') | PAM(5'-3') |
| --- | --- | --- |
| crEGFR-1# | AGGCCAAGCCTGAATCAGCAAAA | TTTC |
| crEGFR-2# | AGAATATCCAGTTCCTGTGGATC | TTTC |
| crEGFR-3# | AAGACTCACTCTCCATAAATGCT | TTTA |
| crPLK1-1# | CATGGACATCTTCTCCCTCTGGT | TTTC |
| crPLK1-2# | GGTGCAGGTACTGGCAGCCAAGC | TTTC |
| crPLK1-3# | GACTGGCAACCAAAGTCGAATAT | TTTG |
| crScramble | TTTTGTACTACACAAAAGTACTG | n.a |

Table S3. oligonucleotides for crRNA *in vitro* transcription.

| name | Target Sequences (5’ to 3’) |
| --- | --- |
| T7-crEGFR-1 F | TAATACGACTCACTATAGGGAATTTCTACTCTTGTAGATAGGCCAAGCCTGAATCAGCAAAA |
| T7-crEGFR-1 R | TTTTGCTGATTCAGGCTTGGCCTATCTACAAGAGTAGAAATTCCCTATAGTGAGTCGTATTA |
| T7-crEGFR-2 F | TAATACGACTCACTATAGGGAATTTCTACTCTTGTAGATAGAATATCCAGTTCCTGTGGATC |
| T7-crEGFR-2 R | GATCCACAGGAACTGGATATTCTATCTACAAGAGTAGAAATTCCCTATAGTGAGTCGTATTA |
| T7-crEGFR-3 F | TAATACGACTCACTATAGGGAATTTCTACTCTTGTAGATAAGACTCACTCTCCATAAATGCT |
| T7-crEGFR-3 R | AGCATTTATGGAGAGTGAGTCTTATCTACAAGAGTAGAAATTCCCTATAGTGAGTCGTATTA |
| T7-crScrambled F | TAATACGACTCACTATAGGGAATTTCTACTCTTGTAGAT TTTTGTACTACACAAAAGTACTG |
| T7-crScrambled R | CAGTACTTTTGTGTAGTACAAAAATCTACAAGAGTAGAAATTCCCTATAGTGAGTCGTATTA |
| T7-crPLK1-1 F | TAATACGACTCACTATAGGAATTTCTACTCTTGTAGATCATGGACATCTTCTCCCTCTGGT |
| T7-crPLK1-1 R | ACCAGAGGGAGAAGATGTCCATGATCTACAAGAGTAGAAATTCCTATAGTGAGTCGTATTA |
| T7-crPLK1-2 F | TAATACGACTCACTATAGGAATTTCTACTCTTGTAGATGGTGCAGGTACTGGCAGCCAAGC |
| T7-crPLK1-2 R | GCTTGGCTGCCAGTACCTGCACCATCTACAAGAGTAGAAATTCCTATAGTGAGTCGTATTA |
| T7-crPLK1-3 F | TAATACGACTCACTATAGGAATTTCTACTCTTGTAGATGACTGGCAACCAAAGTCGAATAT |
| T7-crPLK1-3 R | ATATTCGACTTTGGTTGCCAGTCATCTACAAGAGTAGAAATTCCTATAGTGAGTCGTATTA |
| T7-crE-P F | TAATACGACTCACTATAGGGTAATTTCTACTCTTGTAGATAGGCCAAGCCTGAATCAGCAAAATAATTTCTACTCTTGTAGATGACTGGCAACCAAAGTCGAATAT |
| T7-crE-P R | ATATTCGACTTTGGTTGCCAGTCATCTACAAGAGTAGAAATTATTTTGCTGATTCAGGCTTGGCCTATCTACAAGAGTAGAAATTACCCTATAGTGAGTCGTATTA |

Table S4. pre-crRNA arrays for *in vitro* cleavage reactions

AATTTCTACTCTTGTAGATCTGATGGTCCATGTCTGTTACTCAATTTCTACTCTTGTAGATTGGTTGCCCACCCTAGTCATTGGAATTTCTACTCTTGTAGATCTAGGAATATTGAAGGGGGCAGGAATTTCTACTCTTGTAGATGTGCTCAATGAAAGGAGATAAGG

Direct repeat sequences (DR) are underlined

Table S5. PCR primers for amplification of DNA regions for T7E1 assay

| name | Sequences (5’ to 3’) | cleavage band |
| --- | --- | --- |
| EGFR Sur F1 | gctttggctgtggtcaactt | 100+311bp |
| EGFR Sur R1 | GCCTCTTCGGGGTAATCAGA |  |
| EGFR Sur F2 | cccaagcctggacttaacgt | 130+283bp |
| EGFR Sur R2 | CTCCATCAGTGGCGATCTCC |  |
| EGFR Sur F3 | CTGAACCTGTGACTCACCCC | 100+331bp |
| EGFR Sur R3 | AAGCGGCCCATGGGAAATAA |  |
| PLK1 Sur F1 | CTCCACTTCACTGGAACCCC | 186+338bp |
| PLK1 Sur R1 | CTCCACCGGCGAAAGAGATC |  |
| PLK1 Sur F2 | GAGAAGGGGTGCTGCGAATG | 115+386bp |
| PLK1 Sur R2 | GACTTGTGGGTTGTCTCCTTCCT |  |
| PLK1 Sur F3 | GACTGGCAACCAAAGTCGAATAT | 120+347bp |
| PLK1 Sur R3 | CCTCCTCCGCTGTACCCATG |  |

Table S6. Primers used in qRT-PCR

| Primer names | Forward (5’-3) | Reverse (5’-3) |
| --- | --- | --- |

| Gapdh | | TTGATGGCAACAATCTCCA | ACAACCTGAGGGGAAAGTCC | | |
| --- | --- | --- | --- | --- | --- |
| Il-1β | TGTGTAATGAAAGACGGCA | | | TCCACTTTGCTCTTGACGGCAC |  |
| Il-6 | AGCCAGAGTCCTTCAGAGAG | | | CTTAGCCACTCCTTCTGTGAC |  |
| Tnf-α | CAAAATTCGAGTGACAAGCCT | | | CTGGGAGTAGACAAGGTACAAC |  |
| IL-6-F | | AGCCAGAGTCCTTCAGAGAG | CTTAGCCACTCCTTCTGTGAC | | |
| IL-1β-F | | TGTGTAATGAAAGACGGCA | TCCACTTTGCTCTTGACGGCAC | | |
| TNF-a-F | | CAAAATTCGAGTGACAAGCCT | CTGGGAGTAGACAAGGTACAAC | | |
| LAMA5 | | GCTGTGTGAAGAGACTGAGG | CCTGGGAGGTCTAAAGTGAT | | |
| TNXB | | GGACTCCATGAGCTACCACA | TAGTGGCAGTTCCTGTACCA | | |
| IL7R | | CTGAGGATGTAGTCATCACT | TGTAGTCCCAAGGCTAAGCA | | |
| STAT3 | | ACCAGCAGTATAGCCGCTTC | GCCACAATCCGGGCAATCT | | |

**Table S7**. **Primers used in targeted deep sequencing**. **List of putative off-target sequences of crEGFR and crPLK1.** Nucleotide mismatch are shown in lowercase. PCR primers for identified potential off-target genes. MM, mismatch.

|  | Name | Targets equence(23nt) | MM | Locus | primers (Forward and Reverse, 5'-3') | Product Size（bp） |
| --- | --- | --- | --- | --- | --- | --- |
| crEGFR | On-target | AGGCCAAGCCTGAATCAGCAAAA | 0 | chr7: 55157666 | 5'-CCCTGCGCATGTACACTCAG-3'  5'-CAAAGGCATGGAGGTCCGTC-3' | 221 |
| Off-targets of crEGFR | OT-1 | AGGCCAAcaaTcAATCAGCAAAt | 4 | chr8: 41323023 | 5'-GGTGCAGGATGGCTCACATT-3'  5'-TAGAGGAGGGCGTGTGTATGA-3' | 249 |
|  | OT-2 | AGGCCAtGaaTGAATCAGagAAA | 5 | chr8: 102030230 | 5'-CTTTCTATAGAGGGTGTGCCCC-3'  5'-TTGACCAGTGGAAGCCTTTGA-3' | 203 |
|  | OT-3 | AGGaCAAGgCTtAAaCAcCAAAA | 5 | chr5: 123064258 | 5'-AGGCCAATAGCGGTTGCTTA-3'  5'-TTGTCTGAGGTCCCATTGCT-3' | 230 |
|  | OT-4 | gtcCCAAGCCTGAtTCAGgAAAA | 5 | chr5: 158975165 | 5'-CCATCAGCTCTGGTCACTGAT-3'  5'-GGAGGTGTAGGAGACTGGGTT-3' | 200 |
|  | OT-5 | AGGCtAAGCCTGAgTgtGCAgAA | 5 | chr1: 78201160 | 5'-TGTTGTGGTCCTAATGTCCTTTT-3'  5'-GCCCACAGCTCTTGTTGTCA-3' | 234 |
|  | OT-6 | ctGCCAgGCCTGAgTCAGCAAAA | 5 | chr1: 113166553 | 5'-CTGTTCGGTAAAACTGCGTCT-3'  5'-CTTGACTTGTGGCACTGATCC-3' | 207 |
|  | OT-7 | AGagaAAGtCTGAATCAGtAAAA | 5 | chr1: 195406653 | 5'-AGGCTGAACAGAGCTCCATAA-3'  5'-GGTCTCGGTCTTGTCTATGGG-3' | 244 |
|  | OT-8 | AGGtCAAaCCTGAtTCctCAAAA | 5 | chr7 : 21368584 | 5'-ACAAGTTGAACCCTCAGCCT-3'  5'-GACTGATTTGACCCCCTTAAAGT-3' | 247 |
|  | OT-9 | AGatCAAGCtTcAATgAGCAAAA | 5 | chr12: 67566532 | 5'-TCCAGTGCTTTGCGTGAAGA-3'  5'-TCTGAAGTTGGAGAAATTGTTG TCA-3' | 200 |
|  | OT-10 | AGGaCAAaCgTtAATCAGCtAAA | 5 | chr21: 41123126 | 5'-CAACACGACCACTGTCTCCT-3'  5'-AAGGGTTGGCAACTAGTGCTG-3' | 200 |
| crPLK1 | On-target | GACTGGCAACCAAAGTCGAATAT | 0 | chr8: 62144170 | 5'-GGAAGATTCCTGGGCAAGCC-3'  5'-TGCCCTTTCTTGCTCAGCAC-3' | 262 |
| Off-targets of crPLK1 | OT-1 | atCTGGtAAgCAAAGTCaAATAT | 5 | chr5: 123370987 | 5'-TCCCCAATGTTACTTTCTGA TTACA-3'  5'-TGGATGAGAGATGCTGAA CCTG-3' | 236 |
|  | OT-2 | GggTGGaAtCCAAAGTCaAATAT | 5 | chr1: 160573277 | 5'-CTTCAAGGCTGGGCAAGCAA-3'  5'-GGCAGAGCCTAGTAGTGATGG-3' | 202 |
|  | OT-3 | aAgTGGCAAgCAAAaTCGAATta | 5 | chr2: 100089403 | 5'-AAGGGGGACTGATGAAGAAAGT-3'  5'-TTGCCTGGTTCCCTTCCAATA-3' | 247 |
|  | OT-4 | aAgTGGgAAgCATAGTCGAtTtT | 5 | chr2: 109775792 | 5'-AAGGGGGACTGATGAAGAAAGT-3'  5'-TTGCCTGGTTCCCTTCCAATA-3' | 247 |
|  | OT-5 | GAaTGGgAAaCAAAtTgGAATAT | 5 | chr2: 190160554 | 5'-CCCTACTTCAAAATGCCAA ACCC-3'  5'-TCAGTGTAGGTCAAAAGTCT AGTCA-3' | 247 |
|  | OT-6 | GcCTGGCAACtAAAGTactATAT | 5 | chr12: 77512661 | 5'-GTGTTGCAGATTTCCAGCCA-3'  5'-GCGCAGTCAAAAGGAAAGCAT-3' | 222 |
|  | OT-7 | GACTtcCAgCtAAAGTtGAATAT | 5 | chr3: 97849390 | 5'-AGGCAGGGAGTTCTCCAAGTG-3'  5'-CCTCAAGCTGGGCAACTTTTTC-3' | 214 |
|  | OT-8 | GACTGtCAACtAAAtTtaAATAT | 5 | chrX: 22315784 | 5'-TAGGTCAGCGCTCAAAAGAA-3'  5'-GGAAAATCAAAAGGCCCAAAGG-3' | 249 |
|  | OT-9 | GgCTGGCAAgCAAAGagGgATAT | 5 | chr11: 122109079 | 5'-TCAGTCTAGGTTCCCTGCCA-3'  5'-CCCAACCACCAGGCTATCTG-3' | 202 |
|  | OT-10 | tACTaGtAACCAAAGTtGAAcAT | 5 | chr18: 24327785 | 5'-TCAGAAGATGCCATTCTGACTT-3'  5'-TGTAGATTCAGAAGTGTCCAA ATGA-3' | 219 |
